# Gut-microbiota in children and adolescents with obesity: inferred functional analysis and machine-learning algorithms to classify microorganisms

**DOI:** 10.1038/s41598-023-36533-2

**Published:** 2023-07-12

**Authors:** Margherita Squillario, Carola Bonaretti, Alberto La Valle, Eddi Di Marco, Gianluca Piccolo, Nicola Minuto, Giuseppa Patti, Flavia Napoli, Marta Bassi, Mohamad Maghnie, Giuseppe d’Annunzio, Roberto Biassoni

**Affiliations:** 1 IRCCS Ospedale Policlinico San Martino Genoa, Genoa, Italy; 2 Molecular Diagnostics, Analysis Laboratory, IRCCS Istituto Giannina Gaslini, Genoa, Italy; 3 Pediatric Clinic, Regional Center for Pediatric Diabetes, IRCCS Istituto Giannina Gaslini, Genoa, Italy; 4 Department of Neurosciences, Rehabilitation, Ophthalmology, Genetics, Maternal and Child Health, (DINOGMI), Università degli Studi di Genova, Genoa, Italy; 5 Neuro-Oncology Unit, IRCCS Istituto Giannina Gaslini, Genoa, Italy; 6 Department of Pediatrics, Pediatric Clinic, Regional Center for Pediatric Diabetes, IRCCS Istituto Giannina Gaslini, Via Gaslini 5, 16147 Genoa, Italy

**Keywords:** Biological techniques, Biomarkers, Diseases, Endocrinology, Medical research

## Abstract

The fecal microbiome of 55 obese children and adolescents (BMI-SDS 3.2 ± 0.7) and of 25 normal-weight subjects, matched both for age and sex (BMI-SDS − 0.3 ± 1.1) was analysed. Streptococcus, Acidaminococcus, Sutterella, Prevotella, Sutterella wadsworthensis, Streptococcus thermophilus, and Prevotella copri positively correlated with obesity. The inferred pathways strongly associated with obesity concern the biosynthesis pathways of tyrosine, phenylalanine, tryptophan and methionine pathways. Furthermore, polyamine biosynthesis virulence factors and pro-inflammatory lipopolysaccharide biosynthesis pathway showed higher abundances in obese samples, while the butanediol biosynthesis showed low abundance in obese subjects. Different taxa strongly linked with obesity have been related to an increased risk of multiple diseases involving metabolic pathways related to inflammation (polyamine and lipopolysaccharide biosynthesis). Cholesterol, LDL, and CRP positively correlated with specific clusters of microbial in obese patients. The Firmicutes/Bacteroidetes-ratio was lower in obese samples than in controls and differently from the literature we state that this ratio could not be a biomarker for obesity.

## Introduction

During the past decades, the prevalence of childhood obesity has dramatically increased worldwide. In most developed countries (the U.S. at first), being overweight or obese is the most common chronic disease in childhood and adolescence and represents a serious public health problem. It has been reported that obesity affects nearly 107.7 million children and adolescents worldwide^1^. Several factors have been identified as potential risk factors for pediatric obesity and type 2 diabetes: early nutritional and epigenetic mechanisms, maternal malnutrition and microbiota assessment^2^.

The adverse consequences of obesity include several conditions, like insulin resistance and type 2 diabetes mellitus^3^. Both increase the risk for cardiovascular and cerebrovascular morbidity and mortality, which shorten life expectancy.

Different articles describe the microbiome of obese children and adolescents, sometimes with conflictual results. These articles are rather heterogeneous on the procedures for studying the microbiome, ranging from culture technique and PCR targeting only a limited number of taxa to Next-Generation sequencing (NGS)^4–7^. Moreover, it is known that the microbiome could be affected by different drugs such as Metformin and Liraglutide^8,9^. Most articles used on NGS are based on the PCR amplification of a single polymorphic region of the 16S-ribosomal subunit gene. Part of the generated conflicting results may also depend on the variegated criteria used to classify the obese population enrolled in the analysis. Indeed, previously published results are based on adult or pediatric populations considering a mix of obese and overweight patients selected based on BMI alone or BMI z-score, but few on BMI-SDS in pediatric cohorts. In the present study, the fecal microbiome was assessed using NGS technology, and the children/adolescent patients were grouped based on BMI-SDS. Since it is known that the analysis of a single or of a couple of 16S-hypervariable regions did not give an exhaustive representation of the microbiome^10^, we opted for the NGS-sequencing of 7 out of the 9 polymorphic 16S-regions.

## Results

### Microbiome analysis and the *Firmicutes/Bacteroidetes* ratio

The 16S-mapped reads obtained from all analyzed microbiome samples were 211,854 ± 56,420 and resulted in the identification of 1797 ± 400 operational taxonomic units (OTUs) in the simple obese patients (OB-G), 1761 ± 509 OTUs in the obese with complications (OBc-G) samples and 1747 ± 602 OTUs in normal weight Healthy Donors (nwHD) used as controls. A visual analysis of the *Firmicutes* and the *Bacteroidetes* phyla abundances and their *Firmicutes/Bacteroidetes* (F/B) ratio indicated that the samples from obese patients had always a lower F/B-ratio value than the group of nwHDs, indicating that F/B ratio is not a biomarker for obesity, differently from what proposed by some authors^11,12^. These data are confirmed both by the analysis of the consensus of seven 16S-polymorphic regions (F/B-ratio: Obese:1.2 ± 0.8 vs nwHD:1.6 ± 1.8, respectively) and by analyzing the V4-region alone (F/B-ratio: Obese:0.9 ± 0.7 vs nwHD:1.2 ± 1.3, respectively). It is to be stressed that the V4 region is the most studied 16S polymorphic region reported in the literature on metagenomics.

### Alpha and beta diversity analysis

Different alpha diversity profiling indices have been used that estimate either the community richness (Chao1-index) or richness and evenness (Shannon or Simpson indexes). It is important to highlight that none of the analyzed alpha diversity indexes reached statistical significance in the comparison between the complete case series of obese patients (55 fullOB) and the 25nwHD. In detail, the trend of Chao1 mean values were slightly higher in pathological samples (143.2 ± 22.3) rather than in nwHDs (141.5 ± 30.1) (Supplementary Fig. 1a). Simpson (Si) and Shannon (Sh) indexes behaved in the opposite way, where both were slightly higher in nwHDs (Si: 0.946 ± 0.014–Sh: 5.057 ± 0.320) rather than fullOB (Si: 0.87 ± 0.10–Sh: 2.83 ± 0.45) (Supplementary Fig. 1a). The Bray–Curtis index used for beta diversity analysis clearly showed statistical significance (PERMANOVA p < 0.02) in the comparison between fullOB and nwHDs, thus indicating a difference in microbiome composition between patients and normal weight controls (Supplementary Fig. 1b).

### Comparative analysis: fullOB*vs*nwHD

**Figure 1:**
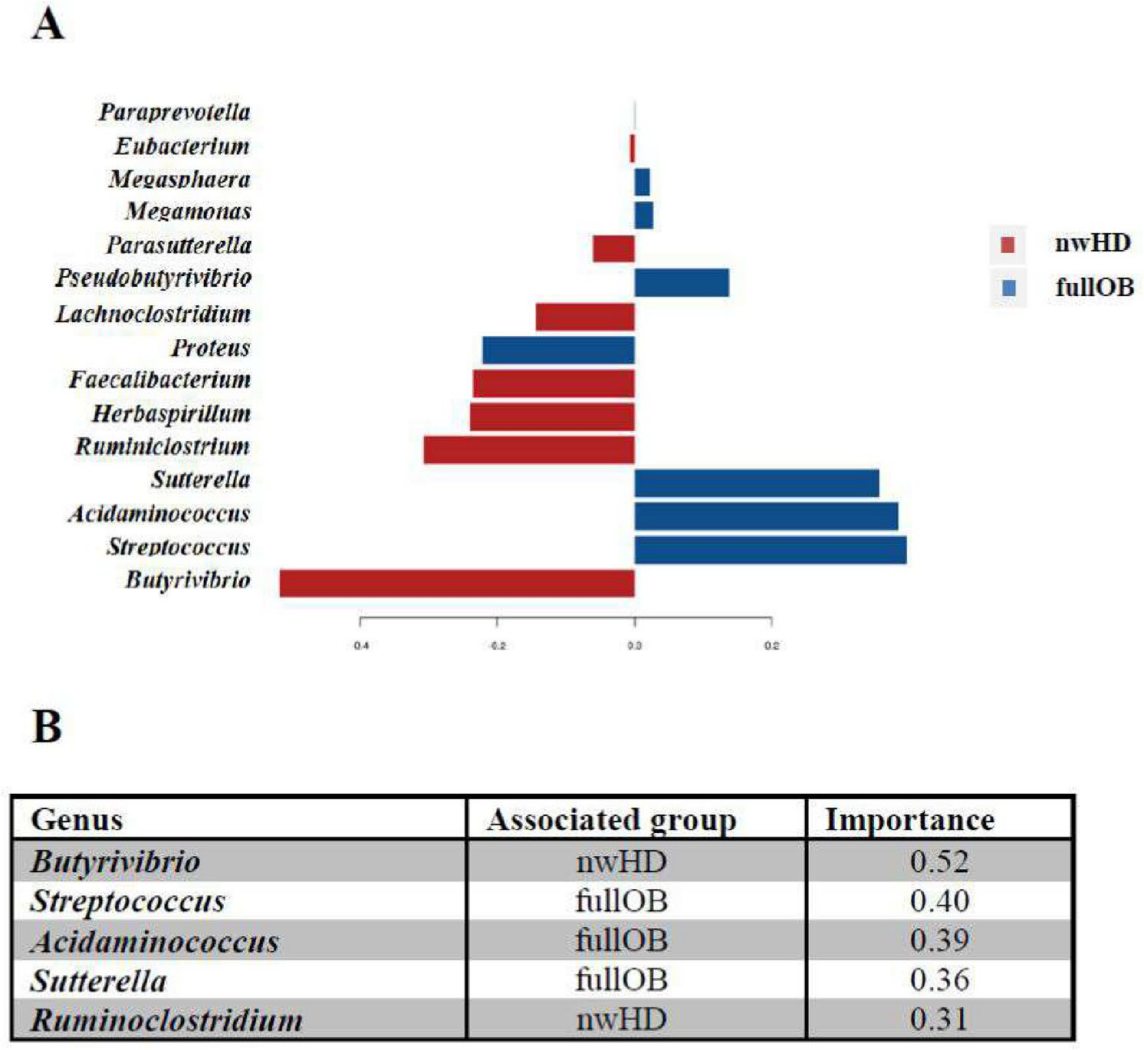
The sPLS-DA results between fullOB (blue) *vs* nwHD (red) associated fecal microbiome. Loading plot from the sPLS-DA applied to the data to discriminate in the microbiome the Obese (fullOB) patient's associated taxa from the ones linked to controls (nwHD). Colors indicate the classes in which the median is maximum for each significant taxa (red) for regular weight Healthy Donors (nwHD) and blue for Obese (fullOB). The negative and positive values indicate positive and negative associations (importance) identified among the statistically significant identified taxa.

**Figure 2:**
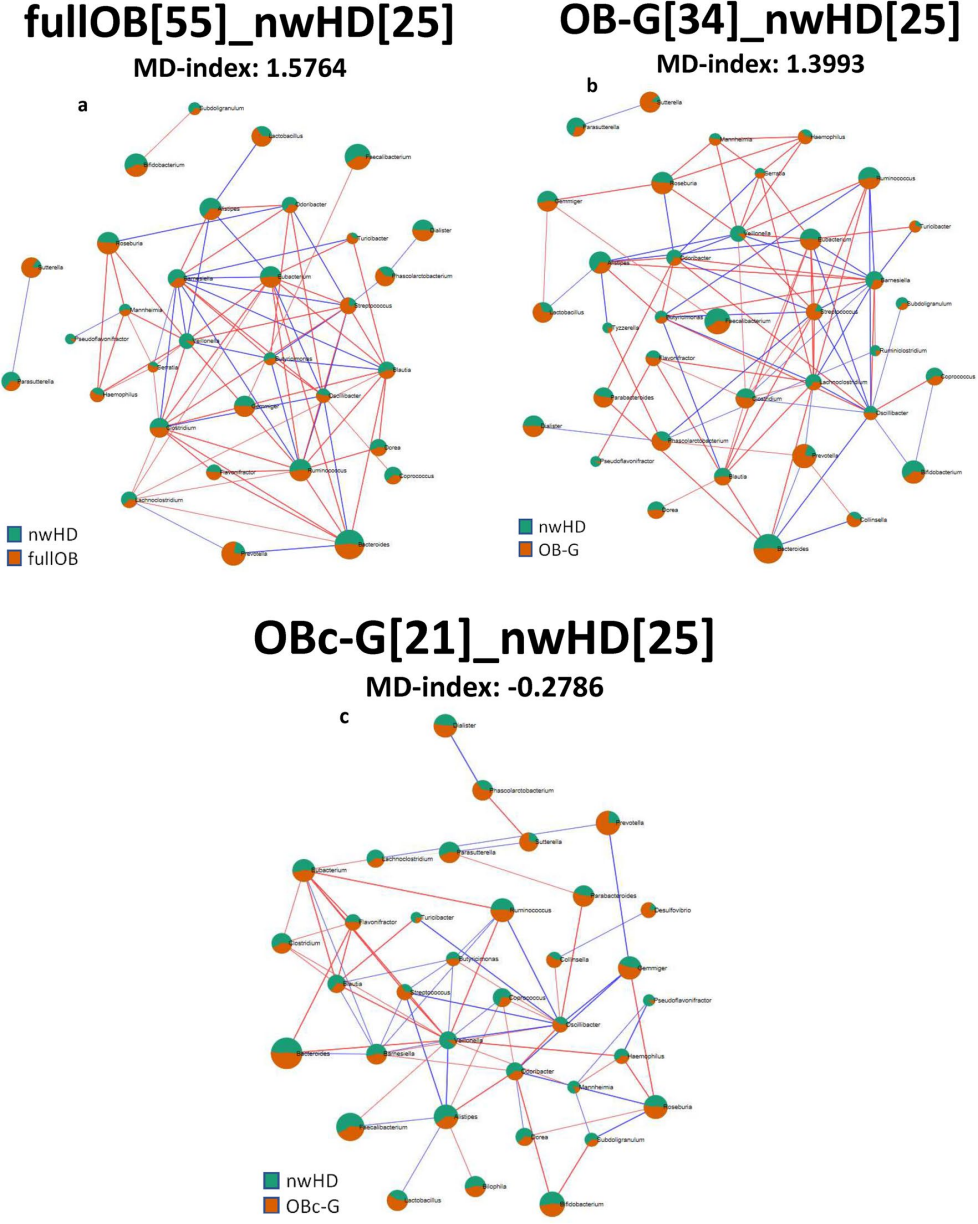
SparCC correlation networks. Taxa are connected by an edge when their correlation meets the p-value (< 0.05) and the correlation thresholds (0.3). The edge size reflects the magnitude of the correlation. These networks show significant positive (red edges) or negative (blue edges) Pearson correlations. The size of the rounded area for each node represents the abundance of that taxon, and the colors show the proportion of the associated group. (**a**) Green for normal-weight Healthy Donors (25 subjects) and orange for the complete case seriesof obese patients (55 cases). The MD-index was 1.5764, computed at the genus level for comparing the microbiome of obese patients over the normal weight controls. (**b**) Green for normal-weight Healthy Donors (25 subjects) and orange for the obese patients (34 cases) with no additional complication. The MD-index was 1.3993. (**c**) Green for normal-weight Healthy Donors (25 subjects) and orange for the obese with complication patients (21 cases). The MD-index was − 0.2786. In the analysis of the microbiome of plain obese patients (OB-G) compared with the microbial flora present in controls, there is a slightly unbalanced overrepresentation of some genera in the Obese samples rather than in control subjects. Whereas obese patients with complications showed an unbalance due to an underrepresentation of some genera confronted with the same population of controls (nwHD). Note that the MD index of a Eubiotic state is equal to 1.

The specific associations between taxa present in fullOB and nwHD were analyzed using the Calypso^13,14^ package which considers the sparsity (i.e., a dataset with many values equal to zero) and the compositional origin of microbiome data. Specifically, we used the sparse Partial Least Squares-Discriminant Analysis (sPLS-DA)^14^ that associates the importance of a specific taxon to describe a group of samples (Fig. 1). The results indicated that among the first 5 genera ordered for importance, 3 of them showed a positive correlation with fullOB (*Streptococcus, Acidaminococcus and Sutterella* with importance scores of 0.40, 0.39, and 0.36, respectively). The relative abundance analysis, using 4 different algorithms within MicrobiomeAnalyst, and the sparse correlation for compositional data (SparCC) analysis confirmed this result (Fig. 2a and Supplementary Table 1). A positive correlation with fullOB was found for *Sutterella wadsworthensis* and *Streptococcus thermophilus*, both characterized by 10 times higher abundances in obese patients compared to nwHD (EdgeR log_2_ fold change values 3.4795 and 3.7707 respectively with p-value < 0.01; see Supplementary Table 1). *Prevotella* genus and *PrevotellA* we correlated with full rather than nwHD (Fig. 2a and Supplementary Table 1). The Microbial Dysbiosis index (MD-index) for this comparison is 1.5764 (EdgeR log_2_ fold change values 3.3504 and 3.3511 respectively with p-value < 0.05; see Fig. 2a) indicating a high imbalance (dysbiosis) in the microbial flora of obese patients compared to controls (nwHD).

### Comparative analyses: OB-G *vs* nwHD and OBc-G vs nwHD

The analysis of the more relevant genera using supervised Random-Forest indicated that *Streptococcus* was a genus tightly linked to OB-G, followed by *Sutterella*, *Clostridium*, and *Lactobacillus* (Fig. 2b and Supplementary Table 2). The species that showed a positive association with OB-Group of samples were *Sutterella wadsworthensis* and *Blautiaproducta*(OBB error:0.322; Sensitivity: 0.64 and 0.70 of Specificity; see Supplementary Table 2). Both the *Sutterella* genus and the *Sutterella wadsworthensis* species have confirmed a stronger association with OB-G rather than OBc-G, considering the number of different methods reaching statistical significance and also the result of the relative abundance analysis (Fig. 2b,c. Supplementary Tables 2 and 3 EdgeR log_2_ fold change 3.7642 and 4.6938 with p-value < 0.05 and Supplementary Table 3 EdgeR log_2_ fold change 2.9930 and 4.1985 with p-value < 0.05). The same was true for the *Streptococcus* genus (EdgeR log_2_ fold change 2.6028 with p-value < 0.05)and their descendent species (i.e., *S. australis, S. salivarius* and* S. thermophilus*. EdgeR log_2_ fold change 2.4211, 2.1653 and 5.0708 with p-value < 0.05) that were better described in the OB-Gpatients (Supplementary Table 2). Random-forest's supervised analysis indicated that the genera *Lactobacillus*, *Gemminger *(OBB error 0.413; Sensitivity: 0.61 and 0.56 of Specificity), and the species *Coprococcus comes* and *Bacteroides massiliensis *(OBB error 0.37; Sensitivity: 0.64 and 0.61 of Specificity) showed higher "mean decrease accuracy" and thus a positive association with the OBc-G patients (Supplementary Table 2). More in detail, in relative abundance analysis the *Bacteroides massiliensis* showed a statistically significant value only in the comparison between OBc-G and nwHD (Supplementary Table 3 EdgeRlog_2_ fold change 2.9527 with p-value < 0.05), but not in the comparison between OB-G and nwHD (Supplementary Table 2). On the contrary, *Coprococcus comes* showed a positive association with both OB-G and with OBc-G group of patients (Supplementary Tables 2–3. EdgeRlog_2_ fold change 2.1999 and LDA: 2.16, both with p-value < 0.05).

It is to be noted that some Bacteroides genera behave in opposite ways, indeed *Bacteroides fragilis*, *Bacteroides plebeius*, and *Bacteroides thetaiotaomicron *(EdgeR log_2_ fold change 3.7523, zero-inflated Gaussian fit: 0.0189 and EdgerR log_2_ fold change 2.1903 with p-value < 0.05) showed higher abundances in OB-G patients compared to nwHD. While, on the contrary, *Bacteroides faeces* and *Bacteroides massiliensis *(EdgeR log_2_ fold change 3.1409 and 2.9527 with p-value < 0.05) showed a positive association with OBc-G compared to nwHD (Supplementary Tables 2–3). The MD-index behaves in opposite ways in the OB-G and OBc-G correlation networks with respect to nwHD. Indeed, MD-index computed for OB-G was 1.3993, showing high dysbiosis, while in OBc-G was-0.2786 (Fig. 2b,c, respectively) which still underlinesa dysbiosis but much less evident for OB-G samples.Indeed, in the group of obese patients without any complication (OB-G), there was a slightly unbalanced overrepresentation of some genera concerning the microbiome of control subjects. Whereas in obese patients with complications, some genera were underrepresented concerning controls (nwHD).

### Supervised Random Forest analysis and normal weight healthy donors (nwHD)

We applied the Random Forest machine-learning algorithm^15^ to identify taxa able to discriminate between patients with obesity and normal-weight donors with good classification performances. We already described the correlations between specific taxa and the OB-G or OBc-G. Here we closely analyzed taxa that classify the normal-weight healthy donor group. *Alistipes* genus (Supplementary Tables 1, 2: zero inflated-Gaussian fit of 0.0027 and 0.0015), different *Alistipes* species (Supplementary Table 1: *A. finegoldii, A. sp*. and *A. senegalensis* Edge R log_2_ fold change − 1.4927 and − 1.6049 with p-value < 0.05 and zero-inflated Gaussian fit: 0.0376. Supplementary Table 2: *A. indistinctus* and *A. senegalensis.* EdgeR log_2_ fold change − 2.3560 with p-value < 0.05 and 0.0025 of zero-inflated Gaussian fit. Supplementary Table 4: *A. onderdonkii.* OBB error: 0.262 or 0.319; Sensitivity: 1.00 or 0.65 and 0.72 or 0.77of Specificity), and the *Bifidobacterium longum *(Supplementary Table 1: EdgeR log_2_ fold change − 2.6775 with p-value < 0.01. Supplementary Table 2: EdgeR log_2_ fold change − 2.095 with p-value < 0.05. Supplementary Table 4: OBB error: 0.356 or 0.319; Sensitivity: 0.61 or 0.65 and 0.66 or 0.77 of Specificity) were strongly associated with nwHD. In addition, the genus *Akkermansia* (Supplementary Table 1: zero-inlated Gaussian fit: 0.0025. Supplementary Table 2: OBB error: 0.386; Sensitivity:0.57 and 0.65 of Specificity) and its descendants *Akkermansia muciniphila* (Supplementary Table 1a: zero-inlated Gaussian fit: 0.0376. Supplementary Table 2: OBB error: 0.281; Sensitivity: 0.70 and 0.74 of Specificity) were mainly associated with nwHD (Supplementary Tables 1 and 4). Among *Blautia*species, a different behavior was observed for *Blautiawexlerae* since it appeared closely linked to nwHD rather than obese samples (Supplementary Table 4: EdgeR log_2_ fold change − 3.1199 and p-value < 0.01. Supplementary Table 2: OBB error: 0.281; Sensitivity: 0.70 and 0.74 of Specificity) and other *Blautia*species (*B. faecis* was linked to nwHD see Supplementary Table 1c, while *B. producta* was linked to Overall Severe Obese, see Supplementary Table 2) (Supplementary Tables 3 and 4).

### Correlation analysis of taxa clusters with physiological parameters

**Figure 3:**
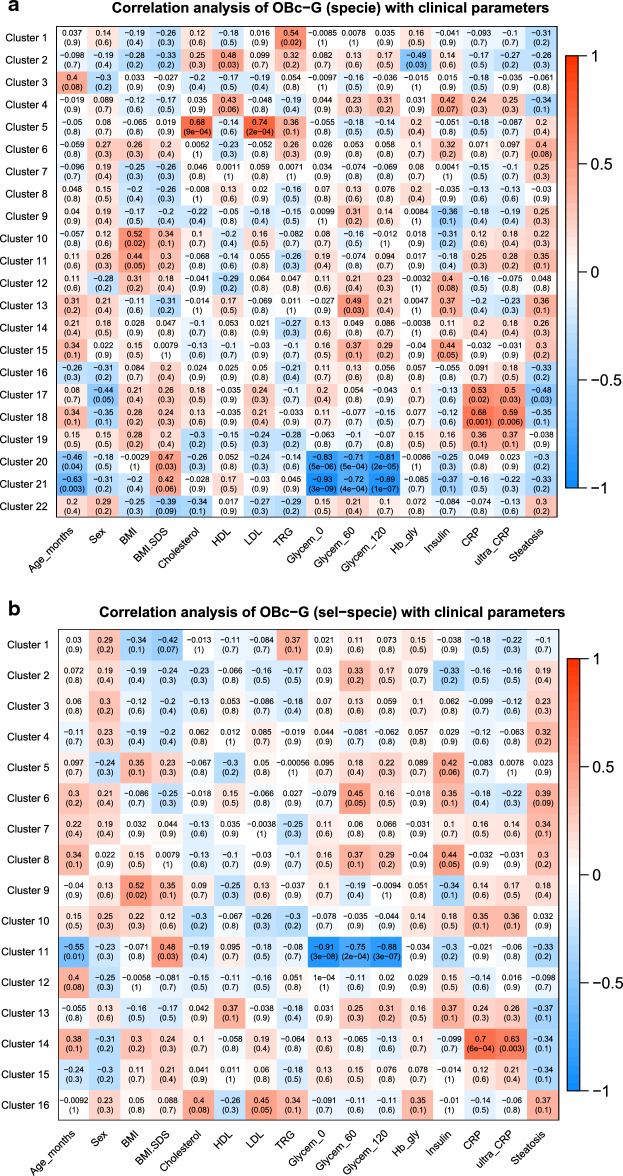

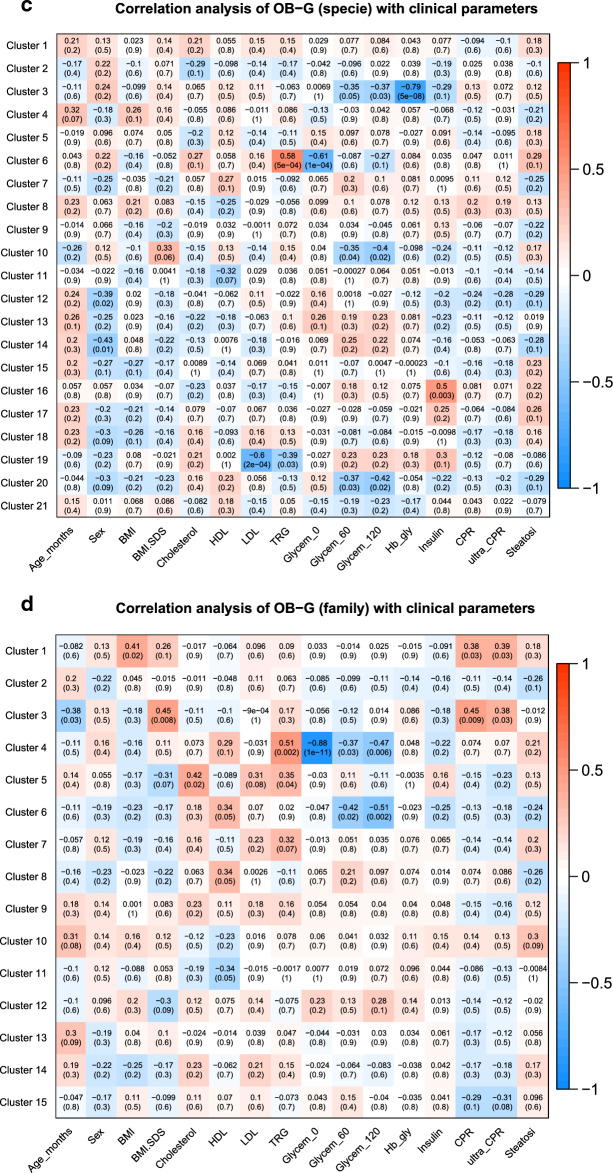

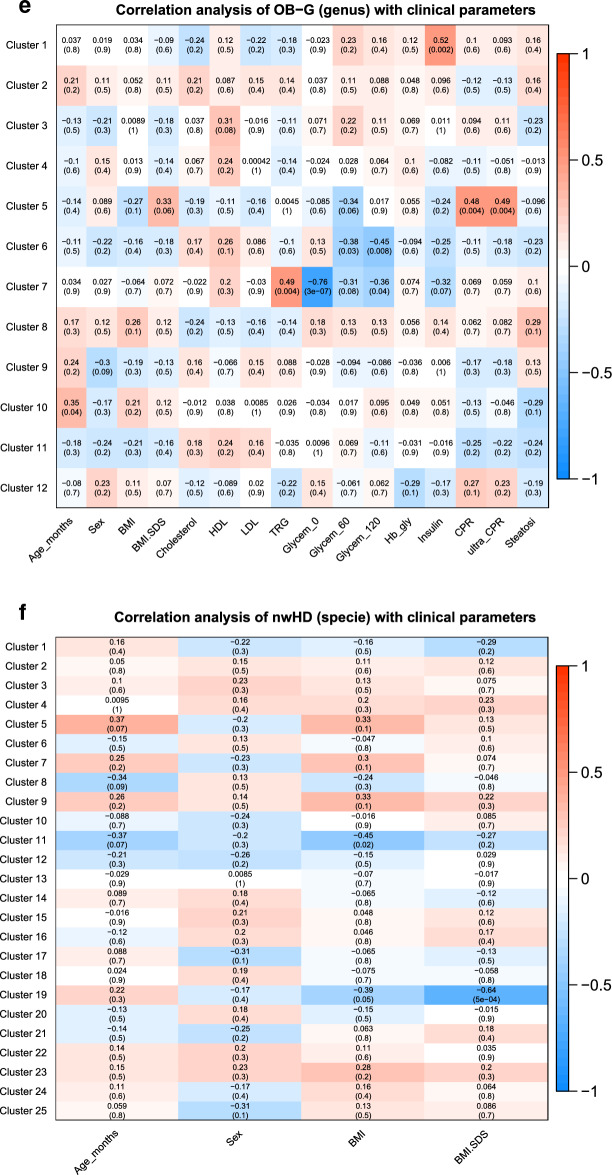
Heatmaps of the correlations between taxa clusters and physiological parametersusing WGCNA. The heatmaps show the results of the multivariate clustering analysis considering the physiological parameters and the unadjusted p-values. The red rectangles highlight those correlations that remained statistically significant after the correction for multiple hypotheses with Benjamini–Hochberg. The colored bar aside from the heatmaps shows the color change associated with different Pearson correlation coefficients: the red color indicates positive correlations while the blue color indicates negative correlations. The “sel-specie” refers to the feature reduction step performed before the WGCNA analysis considering the most relevant specie found in our previous analysis (see “Methods” section).

In the analysis of taxa related to samples of OBc-G, we found interesting positive correlations between some clusters of species and the total cholesterol, LDL levels (Fig. 3a,c and Supplementary Table 5a) and CRP (Fig. 3a,b and Supplementary Table 5a,b). Specifically, the increase of the species enclosed in Cluster 5 is significantly associated with the increase of both total cholesterol and LDL levels (Pearson correlation factor (PC): 0.68 and 0.74 with adj-p-value < 0.05); seemingly the increase of the species enclosed in Clusters 18 (Fig. 3a) and 14 (Fig. 3b), is significantly associated with the increase of CRP, which is a marker of inflammation (PC: 0.59 with adj-p-value 0.04). On the contrary, Glycemia (0′, 60′ and 120′) showed a negative correlation with the species of clusters 20 (PC: − 0.83, − 0.71, − 0.81 with adj-p-values < 0.05), 21 (PC: − 0.93, − 0.72, − 0.89 with adj-p-values < 0.05) (Fig. 3a) and 11 (PC: − 0.91, − 0.75, − 0.88 with adj-p-values < 0.05) (Fig. 3b). In the comparisons of the species analyzed entirely versus the same species analyzed after the feature reduction (Fig. 3a vs. b), we found common species (i) positively associated with CRP and (ii) negatively associated with glycemia (0′, 60′, 120′): specifically, we found (i) *Alistipes indistinctus*, *Clostridium innocuum*, *Desulfovibrio piger* *Prevotella ruminicola *and *Prevotella* in common between clusters 18 and 14, while (ii) *Acidaminococcus fermentans*, *Clostridium cocleatum* and *Clostridium ramosum* in common between clusters 20–21 and cluster 11 (see Supplementary Table 5a,b).While the connection of *Alistipes indistinctus and* *Clostridium innocuum* with obesity or with clinical parameters related to obesity is not known in the literature, *Desulfovibriopiger*, *Prevotella ruminocula* and *Prevotella* species specie are already known to be associated with inflammation, insulin-resistance, hyperglycemia and type 2-diabetes^16^. It is important to highlight that *Desulfovibrio piger* (Clusters 6 for OB-G and Clusters 14, 18 for OBc-G) showed that the increase of the abundance of this species is always associated with the increase in the value of some clinical parameters critical for obesity (such as TRG for OB-G and CRP for OBc-G, respectively).

Interestingly enough, in the clusters of taxa found negatively correlated with glycemia (Fig. 3d,e, and Supplementary Table 5d,e) we found that the family *Oxalobacteraceae* and two genera descending from it, namely *Herbaspirillum* and *Oxalobacter*, were already known to be associated with a decreased of insulin-resistance and glycemia^17^.

Finally, in the analysis performed on the nwHD group, BMI-SDS showed a negative association with five species, enclosed in Cluster 19 (PC: − 0.64 with adj-p-value = 0.05) (Fig. 3f and Supplementary Table 5f). This data strongly suggests that BMI-SDS could represent the most sensible clinical parameter to correctly classify children and adolescents as normal weight, overweight, and obese subjects and that the species belonging to this cluster could be considered protective against obesity.

### Inferring functional (metabolic) pathways characterizing OB-G and OBc-G

**Figure 4:**
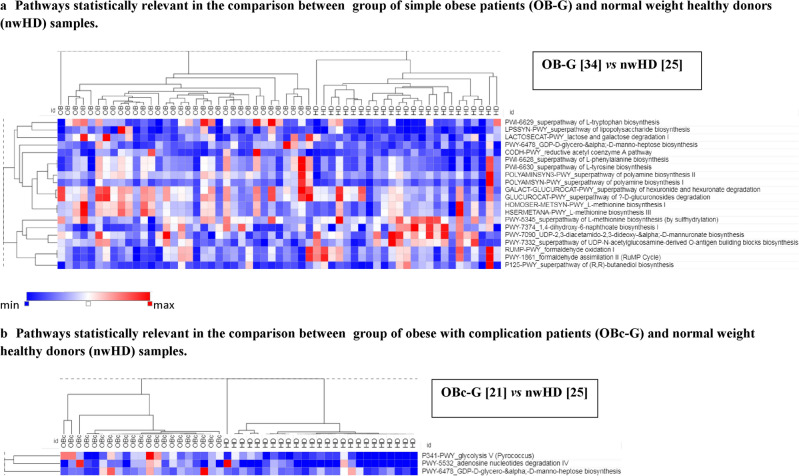
Heatmaps of the significant inferred pathways identified with PICRUSt2. (**a**) Heat-map of the comparisons group of Obese with no complication (OB-G) vs controls (nwHD) and (**b**) group of Obese with complication (OBc-G) vs controls (nwHD).

At least 20 different metabolic pathways, inferred with PICRUSt2 and present in the MetaCycdatabase^18^, showed statistical significance in the comparison between OB-G vs nwHD (Fig. 4a). Different pathways involved in amino acid biosynthesis showed a positive correlation with obesity in pediatric patients. Thus, pathways involving the phenylalanine (PWY-6628) and the tyrosine (PWY-6630) aromatic amino acids were among the entries with the highest statistical importance. In addition, tryptophan (PWY-6629) and methionine biosynthesis (HSERMETANA-PWY and HOMOSER-METSYN-PWY) showed higher abundances in OB-G, while an additional pathway (PWY-5345) for methionine biosynthesis showed an opposite behavior. More, different pathways involved in polyamine biosynthesis known to play a role in bacterial pathogenicity and biofilm formation showed higher abundances in the microbiome of OB-G. Among them, the POLYAMINSYN3-PWY is the one showing the higher importance of all pathways analyzed by Random-Forest analysis (mean decrease accuracy 0.006). Others like POLYAMSYN-PWY and the pro-inflammatory lipopolysaccharide^19^ pathway (LPSSYN-PWY) showed higher abundances in OB-G compared to lean subjects. More, the second in order of importance in the Random-Forest analysis (mean decrease accuracy 0.004) was the super-pathway of (R,R)-butanediol biosynthesis (P125-PWY) which was overabundant only in nwHDs.

Obesity in adolescents is often associated with clinical complications such as insulin resistance, hyperglycemia, dyslipidemia, and hypertension, which together are termed "metabolic syndrome". Regarding this issue, we found 3 pathways more abundant in OBc-G with respect to nwHD: PWY-341 (glycolysis V), PWY-5532 (adenosine nucleotides degradation), and PWY-6478 (GDP-d-glycero-alpha-d-manno-heptose biosynthesis) (Fig. 4b). The heptose-sugars are components of bacterial cell surface common in the pro-inflammatory lipopolysaccharide (LPS)^20^.

## Discussion

Microbiome studies might be hampered by different technical problems related to the methods used for their analysis^21^ not evident to the average readers. The 16S gene is structured in nine variable regions useful to define microbial taxonomy^22–24^. Primer pairs design is to hybridize in the conserved sequence regions, so it is clear that the choice of their sequence directly influences the taxa composition of the microbiome under analysis. Less important but still relevant in the microbiome definition might be the use of different 16S-ribosomal sequence reference databases. More, additional parameters show only marginal effects in the resulting microbiome composition. Indeed methods of clustering the 16S sequences, bioinformatic pipeline, and the parameters used in the analysis might slightly modify the microbiome composition^21^. All these issues might be potential biases to complicate the comparison of microbiome biomarker (taxa or Firmicutes/Bacteroidetes-ratio) composition in different publications, even for the same pathology. Due to the not ideal primer pairs design that usually targets a single 16S variable region, it is likely to have bacterial taxa in the analyzed microbiome might be under-represented. Thus, to overcome these problems, a possibility is to increase the number of the variable regions to be studied and to use different 16S-ribosomal sequences reference databases, bioinformatic pipelines, and parameters, thus improving the definition of biomarkers associated with the pathological microbiome. Our aim was not only an academical re-analysis of taxa associated with obesity but with the introduction of the analysis of multiple 16S-variable regions in the microbiome analysis together with the use of rarely used bioinformatic pipelines and parameters try to define which biomarkers were still associated with the obese patients. We believe that comparing the biomarkers defined using different primer pairs, chemistry, and methods with the ones already shown in published reports indicates the taxa that are more strictly associated with the pathology.

The relative abundance analysis using the complete cohort of fecal samples of obese patients (fullOB) compared to nwHD confirmed that *Acidaminococcus, Sutterella, Streptococcus*, *Prevotella, Lactobacillus,* and some *Bacteroides* species correlated with obesity, as shown by different published articles (see a meta-analysis)^25^. Indeed, the *Acidaminococcu*s genus was reported to be significantly associated with obesity in adult Hispanic subjects^26^ and with pro-inflammatory diets^27^. In our data, *Acidaminococcus* was better associated with the group of patients with Severe Obesity (OB-SO-G) (Supplementary Table 4). Therefore, it was not astonishing that others have found such an increase in patients with type 2 diabetes (T2D)^28^. *Sutterella* genus was already described to correlate positively with obesity in obese Chinese children^29^, but others found the opposite in adults^30^. In line with this data, *Sutterella wadsworthenis* was reported to be positively associated with insulin resistance^31^. Our data showed taxa strongly associated with the microbiome of Obese patients (fullOB, OB-G, and OBc-G) in the comparisons with the fecal flora of normal weight control subjects (nwHD) (Supplementary Tables 1–4).

*Streptococcus* genus was already shown to be correlated in adult cases with BMI^32^. In this study, Streptococcus descendant like *S.thermophilus* was found to be associated with obesity also in our data. These also indicated a positive *Prevotella*association between and its descendants in obesity. Indeed, the role of the *Prevotell* and Prevotella *copri* in obesity is still debated since beneficial and detrimental roles in health have been described for both taxa^33,34^. *Prevotellaceae* and the genus *Prevotella* have been associated with inflammation and insulin resistance^25,35^. It is also of note that *Prevotellacopri*showed to be associated with an altered glucose metabolism leading to glucose intolerance and reduced insulin sensitivity due to the presence of the LeuBgene^31^. It is also known that *Bacteroides* and *Prevotella* genera have a negative correlation with serum leptin levels and a positive correlation with GHrelin serum levels^36^. Since leptin is known to inhibit hunger while GHrelin increases the drive to eat, the effect of these taxa on obesity is consistent^36^. Indeed, the leptin sensitizer butendiol (produced in P125-PWY pathways) was found to have a lower abundance in our simple-obese patients than in controls. Thus, the mechanism inducing obesity relative to the P125-PWY might induce a less efficient regulation of appetite by leptin^37^.

Obesity has been related to an increased risk of multiple diseases involving oxidative stress and inflammation^38^, and *Prevotella* species have been already described as being more abundant in obese patients with an inflammatory condition^26^. Along this line, in our obese patients with complications, at least ten different species were found to correlate positively with standard CRP levels, a marker of chronic inflammation. Among them, we found *Prevotella ruminicola*, *Prevotella *sp., the genus *Mitsuokella *and *Desulfovibriopiger*. Indeed, *Desulfovibrio* descendant species are known to produce endotoxins and favor alteration of gut permeability leading to the induction of pro-inflammatory responses^39–41^. Thisalso applies to the genus *Collinsella,* which we have shown with greater abundance in severely obese patients^39^. Furthermore, in our data, *Bacteroides* correlated with obesity, as described by others who have found a higher abundance in obesity and a positive correlation with BMI^42^. Some authors have found an inverse correlation between obesity and Bacteroides thetaiotamicron^43^ compared to us. This discrepancy is intriguing since this specie is known to produce high amounts of small-chain fatty acids (acetate and propionate), and the overproduction of acetate is known to induce hepatic de novo lipogenesis and increase adiposity^44^.

Our data showed that descendant species belonging to the *Gordonibacter *genus have a positive correlation with plasma levels of total and LDL cholesterol. Along this line, species belonging to this genus were already known to have a positive association with total cholesterol plasma level^45^.

A recent report showed that different taxa belonging to the Phylum of the Firmicutes were found in the microbiome of adult obese or overweight patients^30^. Also, our data showed that *Firmicutes* descendants *Acidaminococcus* spp.,* Lachnospiraceae*,* Mitsuokella*,* Ruminococcus* spp.,* Streptococcaceae*, and* Streptococcus* are among the taxa that shared higher abundance values in obese patients. One exception is represented by the *Odoribacter* genus, although belonging to *Firmicutes*, which showed a higher relative abundance in normal-weight healthy (nwHD) donors. Indeed, the fact that this genus associates better with nwHDs is not surprising due to the anti-inflammatory potential of this microorganism^46^. Although the Firmicutes/Bacteroidetes-ratio was believed to be a biomarker for obesity^11,12^, its role in this condition was found to be contradictory^47^ since some studies reported a positive correlation between F/B-ratio and the BMI values^48^, others, like our work, found no correlation or showed an opposite trend^48–51^ and essentially a dominance of the Bacteroides genus in obesity^52^. About the inferred metabolic pathways associated with obesity, it is interesting to point out that the abundance of aromatic amino acids (tyrosine, phenylalanine, and tryptophan) has already been reported to be associated with obesity and insulin resistance^48^. In particular, tyrosine was shown to be the more prevalent amino acid associated with insulin resistance in obese children^53^. Tryptophan has also been implicated in the pathogenesis of metabolic disorders such as obesity^54^. Indeed increased levels of tryptophan have been linked to over-nutrition and might be responsible for obesity-related inflammation pathways^55^. Pro-inflammatory conditions are even supported by the pathways LPSSYN-PWY lipopolysaccharide and PWY-6478, both involved in LPS synthesis and assembly^19^. These pathways, along with PWY-341 (glycolysis V), are known to play a central role in promoting a pro-inflammatory environment that supports the production of inflammatory mediators by macrophages, thus contributing to insulin resistance. Furthermore, pyruvate, the final product of glycolysis, is metabolized into acetyl-CoA, which is essential for cholesterol and lipid synthesis^56^. All these conditions are linked to obesity and its complications. In addition, it was shown that the transfer of stools from lean donor recipients into metabolic syndrome patients increased insulin sensitivity of the latter and the abundance of 16 different taxa, including *Oxalobacter formigenes*. This is in line with the negative correlation found between fasting plasma glucose and Taxa Cluster 1, 2, and 3 shown in Supplementary Table 3a^17^.

The main limitation of our study is not many pediatric patients enrolled compared to the number of obese subjects in our region.The major strengths include: (i) the mono-centric recruitment, with strict patients selection; (ii) the use of BMI-SDS to define pediatric obesity (in detail, all overweight patients have been excluded); (iii) similar protocols of selection have been used for classifying regular weight healthy donors; (iv) the analysis of multiple 16S-polymorphic regions to define taxonomy and also (v) the use of different algorithms to analyze the microbiome composition among different groups. Furthermore,it is important to highlight thatour results have not been influenced by the use of drugs known to interfere with microbiota composition, such as metformin and liraglutide^8,9^.

## Methods

### Patients

Patients were stratified into different groups based on the BMI-SDS values obtained comparing WHO growing curves corrected for sex and month age following the references shown https://www.who.int/tools/growth-reference-data-for-5to19-years/indicators. https://www.who.int/tools/growth-reference-data-for-5to19-years/indicators. The patients were defined as overweight if the BMI-SDS values had a standard deviation (SD): >  + 1 SD <  + 2; obese when >  + 2 SD <  + 3, and severely obese SD: >  + 3. Monogenic or syndromic obesity were ruled out in all patients. Thus, we evaluated the fecal-microbiome of a total of 55 obese children and adolescents (fullOB) recruited at Giannina Gaslini Institute in Genoa, Italy, between February 2016, and October 2021 (mean age 13.1 ± 2.9, median 13.0, 36% female; BMI-SDS 3.2 ± 0.7). Inclusion criteria were: Caucasian subjects living in Northern Italy, personal history negative for acute or chronic gastrointestinal diseases, and/or antibiotics or probiotics administration in the previous month. All patients were negative for autoimmune disease screening (i.e celiac and thyroid diseases). Among these fullOB samples, 32 patients were grouped based on BMI-SDS as severely obese (mean age 13.5 ± 3.5, median 13.6, 34% female; BMI-SDS 3.6 ± 0.5), while 22 of them were grouped as obese (mean age 13.2 ± 2.4, median 12.9, 36% female; BMI-SDS 2.6 ± 0.2) (see Tables 1, 2).

**Table 1:** Simple obese (OB-G) patients.

Code	Age	Sex	BMI at onset	BMI-SDS	Obeisty subgroup	Total cholesterol	HDL	LDL	TRG	Glycemia 0'	Glycemia 60'	Glycemia 120	Glicated Hb	Total insulin	CRP	Ultrasensitive CRP	HepaticSteatosis	Metformin therapy	Other clinical infos
5D	12.8	F	37.8	3.39	OB-SO-G	184	40	ND	ND	87	199	174	5.6	1364	0.23	2.22	No	No	Systolic hypertension
6D	8.8	F	31.6	3.62	OB-SO-G	180	35	120	ND	99	142	136	4.5	331	0.61	4.93	Yes	No	
7D	15.8	M	36.3	3.16	OB-SO-G	ND	ND	ND	ND	96	117	118	ND	727	0.23	ND	ND	No	
24D	15.3	F	34.1	2.79	OB-O-G	177	43	110	93	88	92	89	4.87	627	0.23	1.36	Yes	No	Sodium butyrrate
27D	9.8	F	25.2	2.54	OB-O-G	160	70	86	55	95	193	132	5.28	606	0.65	6.25	ND	No	
39D	16.3	F	33.4	2.65	OB-O-G	151	40	95	88	89	89	109	5.03	316	0.23	0.79	Yes	No	
51D	10.2	M	24.6	2.64	OB-O-G	186	66	104	58	90	136	119	5.36	558	0.23	2.83	No	No	
67D	11.1	M	34.3	3.56	OB-SO-G	163	68	97	69	83	104	104	5.04	665	0.67	6.07	No	No	Hepatomegaly
79D	13.8	F	39.8	3.29	OB-SO-G	147	47	92	71	104	183	148	5.32	1166	0.97	9.73	No	Yes	
83D	12.3	M	34	3.29	OB-SO-G	128	47	74	65	98	152	130	5.17	244	0.54	5.11	ND	No	Obesity and asma
85D	8.8	M	34.6	4.4	OB-SO-G	130	58	64	114	95	ND	ND	4.56	ND	0.23	1.3	Yes	No	
108D	10.9	M	29	3.12	OB-SO-G	117	44	71	39	85	113	118	5.35	302	0.23	1.95	No	No	Severe Obesity, Asma and OSAS
120D	16.7	F	33	2.58	OB-O-G	112	38	71	80	115	191	178	5.4	ND	0.23	1.34	No	No	Pre-diabetes with insulin resistance. OSAS—hypercapnia
125D	17.8	F	35.5	2.9	OB-O-G	130	40	72	50	83	92	107	4.96	488	0.23	0.93	No	Yes	Asma. dicoflor therapy. polycystic ovary syndrome (PCOS)
135D	10.6	M	47	4.33	OB-SO-G	127	47	68	80	84	ND	101	4.95	ND	1.42	14.45	No	No	Niaprazine, aripiprazole and depakin therapy
137D	10.4	M	23	2.26	OB-O-G	165	56	104	55	115	169	189	5.75	601	0.23	2.07	No	No	
138D	15.9	M	43.7	3.76	OB-SO-G	182	54	109	99	94	155	135	5.21	804	0.23	1.39	Yes	No	Gallbladder stones
140D	10.6	M	36.2	3.8	OB-SO-G	157	69	79	157	ND	ND	ND	5.43	ND	0.68	6.48	Yes	No	
146D	12.5	M	29.2	2.7	OB-O-G	140	52	79	82	84	135	115	5.4	1128	0.48	4.63	ND	No	Essential hypertension under therapy
148D	17.5	F	44.7	3.83	OB-SO-G	157	53	94	74	86	118	127	5.10	552	0.23	3.28	ND	No	Severe obesity
149D	12.8	M	31.4	2.98	OB-O-G	178	64	108	43	93	ND	ND	ND	ND	0.23	0.71	Yes	No	
151D	14	M	42.2	3.66	OB-SO-G	109	58	53	42	93	131	137	5.19	289	0.47	4.24	Yes	No	Severe obesity and hepatic steatosis
154D	11.4	M	27.2	2.74	OB-O-G	177	42	122	133	88	140	122	5.4	748	0.23	1.5	Yes	No	Celiac. obesity and steatosis
156D	13.2	M	38	3.46	OB-SO-G	113	53	56	46	87	183	139	5.34	2000	0.83	7.88	Yes	No	insulin resistance (2000). Mental retardation, q21.1 duplication
160D	9.8	F	24.9	2.49	OB-O-G	176	65	97	64	89	187	114	5.02	787	0.23	2.07	ND	No	
161D	10.6	F	26	2.48	OB-O-G	172	68	96	50	104	ND	ND	4.74	ND	0.23	0.25	ND	No	Hypertension
162D	12.5	M	39.8	3.65	OB-SO-G	157	38	111	87	85	119	91	5.17	765	0.23	3.33	No	No	Obesity + mild OSAS
168D	11.2	M	50.4	4.24	OB-SO-G	149	46	101	89	94	116	118	4.9	340	1.22	12.3	Yes	No	Severe obesity and hepatic steatosis
170D	12.1	M	37.5	3.57	OB-SO-G	119	61	59	54	102	156	133	4.81	858	0.23	2.8	Yes	No	Severe obesity and RPM. Glucose intollerance. Known chromosome deletion Sigmacillin i.m.therapy
179D	16.2	F	37.9	3.18	OB-SO-G	148	43	104	75	94	119	108	4.26	326	0.23	1.03	ND	No	Depression
180D	5.8	M	26.6	4.94	OB-SO-G	135	41	90	89	104	105	138	5.35	215	1.37	12.38	No	No	Severe obesity and moderate OSAS
184D	16.5	M	43	3.75	OB-SO-G	133	34	88	75	99	134	119	5.5	929	0.9	8.4	Yes	No	Severe obesity and OSAS
186D	9.8	M	29.1	3.45	OB-SO-G	130	33	85	74	87	117	122	5.38	690	0.23	0.75	No	No	Hypertension
195D	14.5	M	27.7	2.25	OB-SO-G	210	39	131	136	97	202	127	4.97	1259	0.23	0.36	Yes	No

**Table 2:** Obese with complication (OBc-G) patients.

Code	Age (years)	Sex	BMI at onset	BMI-SDS	Obeisty subgroup	Total cholesterol	HDL	LDL	TRG	Glycemia 0'	Glycemia 60'	Glycemia 120	Glicated Hb	Total insulin	CRP	Ultrasensitive CRP	Hepatic steatosis	Metformin therapy	Other clinical infos
4V	14.7	F	ND	ND	ND	194	34	ND	ND	82	194	184	5.25	2797	0.61	6	Si	No	
59D	11.3	M	28	2.89	OBc-O-G	188	31	115	219	108	105	129	5.52	221	0.23	ND	No	No	Language and learning difficulties
80D	11.5	F	27.3	2.45	OBc-O-G	176	56	105	108	101	148	138	ND	1404	0.23	0.14	No	No	
84D	14.1	M	30	2.59	OBc-O-G	134	38	85	103	88	204	148	5.02	338	0.23	1.42	Yes	Yes	Macrogol therapy
86D	6.1	F	28	4.28	OBc-SO-G	161	44	103	162	ND	ND	ND	4.64	ND	0.23	0.7	ND	No	
106D	15.2	M	28.6	2.23	OBc-O-G	109	39	68	59	92	165	133	5.4	576	0.23	1.42	Yes	No	Hypertension
109D	13.8	M	28.4	2.42	OBc-O-G	159	41	105	106	91	161	133	6.12	643	0.23	1.39	No	No	
112D	13.7	M	32.8	2.98	OBc-O-G	277	34	210	262	97	121	124	5.88	842	0.23	2.91	Yes	No	
117D	16.4	F	42.8	3.63	OBc-SO-G	161	47	106	115	89	172	152	5.36	861	0.76	6.64	No	No	
118D	13.9	M	29	2.49	OBc-O-G	160	50	91	93	88	192	172	5.79	1869	0.82	7.35	ND	No	Hypertension
119D	16.9	M	30	2.23	OBc-O-G	170	45	99	171	82	232	150	4.83	1746	0.23	0.64	Yes	No	
126D	19.0	F	36.6	3.05	OBc-SO-G	135	34	83	163	89	113	124	4.96	865	0.23	3.56	No	No	Estro-progestinic therapy
141D	13.0	M	26.7	2.3	OBc-O-G	170	38	112	154	94	133	121	5.48	876	0.23	2.3	Yes	No	
153D	14.3	M	42.6	3.68	OBc-SO-G	111	36	66	56	78	112	113	5.62	1117	0.93	8.72	No	Glucophage	Hypertension therapy with amlodipin, GHD
157D	12.8	M	25.2	2.16	OBc-O-G	197	36	116	338	83	154	133	5.82	892	0.23	0.98	ND	No	
166D	14.8	F	41.2	3.51	OBc-SO-G	158	31	115	167	96	172	154	5.11	1892	0.23	2.57	No	No	Hypertension
169D	10.8	F	37.4	3.65	OBc-SO-G	170	40	114	81	100	175	141	5.38	849	0.6	6.51	ND	No	
174D	17	M	38.3	3.34	OBc-SO-G	140	39	101	65	108	158	151	4.96	1085	0.85	7.39	Yes	No	Hypertension
176D	18.0	F	41.4	3.56	OBc-SO-G	192	39	139	151	99	136	110	5.44	625	1.38	11.49	ND	No	
182D	12.8	M	50.6	4.04	OBc-SO-G	175	34	122	120	93	112	127	5.21	168	0.62	6.24	Yes	No	Moderate OSAS
189D	13.0	M	45.3	3.86	OBc-SO-G	148	33	79	229	90	151	140	5.25	1600	0.7	6.3	Yes	No	Mild OSAS

Patients and relevant clinical features: Sex (M:male, F:female), BMI and BMI-SDS at onset, TRG: triglycerids, Obesity group (OB-O-G: Obese in Simple obesity, OB-SO-G: Severe Obese in Simple obesity, OBc-O*G: Obese in obesity with complication, OBc-SO-G: Severe Obese in obesity with complication. CRP (C-reactive protein): mg/dL (< = 0.23:negative value).

We differentiated patients with simple obesity (OB-G), regardless of grade, from patients who had complicated obesity (OBc-G), regardless of grade, but who had at least three out of five of these characteristics HDL < 5th centile, triglyceride values above the 95th centile, blood pressure systolic and/or diastolic above 90° centile, impaired fasting glycemia, impaired glucose tolerance after 2 h from the meal, hepatic Steatosis (see Table 1). It is also noted that patients in the OBc-G have on average a higher ultrasensitive CRP than patients with uncomplicated obesity. More in detail, as reported in Table 1, 34 patients had a diagnosis with simple obesity (OB-Group, OB-G) (mean age 12.6 ± 2.9, median 12.4, 35% female, BMI-SDS: 3.3 ± 0.7), while 21 classify as Obese with complications (OBc-G) (mean age 14.0 ± 2.8, median 13.9, 38% of them of the female gender, BMI-SDS: 3.1 ± 0.7). It is also to be stressed that among the 34 simple-obese (OB-G) patients, 22 were classifiable as severely obese (OB-SO-G) (BMI-SDS: 3.6 ± 0.5), while 12 were obese (OB-O-G) (BMI-SDS: 2.6 ± 0.20). While among the 21 obese with complication (OBc-G) patients, 10 of them were severely obese with complications (OBc-SO-G) (BMI-SDS: 3.7 ± 0.3), and the rest were obese with complications (OBc-O-G) (BMI-SDS: 2.5 ± 0.28) (see Table 1). As a control, we have analyzed the metagenome of fecal samples from 25 normal-weight subjects (nwHD) matched both for age and sex (mean age 12.1 ± 3.0, median 12.7, 40% female, BMI-SDS: − 0.3 ± 1.1).Written informed consent was obtained by patients and caregivers.

Research was performed in accordance with the Declaration of Helsinki.

The study was approved by the local Ethical Committee of Liguria Region (approval letter, enclosed) and by Giannina Gaslini Institute (authorization letter enclosed).

### Fecal microbiota analysis

DNA extraction from fecal samples was performed as reported^57^ and it was used for the 16S amplification reaction performed with Ion 16S™ Metagenomics Kit (Thermo-Fisher Scientific). This method allows the PCR-amplification of 7 out of 9 informative 16S polymorphic regions^58^. Then up to 16 differently bar-coded libraries were automatically loaded onto an Ion-520-chip by the Ion-Chef and sequenced by the GeneStudio-S5-system (Thermo-Fisher Scientific). Data analysis was performed with the Ion-Reporter™ suite (v 5.18.0.2) using the curated-Greengenes (v13.5) and the MicroSEQ ID 16S-rRNA reference library (v2013.1) databases using standard parameters.

### Data analysis

Compositional/functional profiling and comparative-analysis of microbiome data were performed with Microbiome Analyst and Calypso web-tools^13,14,59,60^. All p-values have been adjusted to correct for multiple hypotheses, using Benjamini and Hochberg false discovery rate (FDR < 0.05), unless differently specified. Sparse Correlations for Compositional data (SparCC)^61^ was applied after data-filtering to remove low-quality or uninformative features to study the network of correlation among taxa from the microbiome of obese patients and nwHD controls. In addition, we computed the Microbial Dysbiosis index (MD-index) as the logarithm of the sum of all taxa that increase their abundance over the sum of all taxa that decrease it^57^. WGCNA identified groups of taxa, or modules that were present across a set of clinical conditions, computing a similarity measure, such as Pearson's correlation coefficient, to calculate the relationship between pairs of taxa. These relationships are then used to construct a weighted network of taxa clustered to identify highly interconnected microorganisms, which are assumed to have a similar biological function or to be commonly regulated. For all the analysis Pearson's correlation coefficients were computed and its associated p-values were corrected for multiple comparisons using False Discovery Rate (FDR).The Multivariate clustering methodology based on the weighted correlation network analysis (WGCNA) was used to verify correlations of taxa clusters with the clinical parameters characterizing OB-G,OBc-G and nwHD^62^. In particular, we considered the following list of clinical parameters: sex, age (in months), BMI, BMI-SDS, serum levels of total HDL, LDL and cholesterol, triglycerides (TRG), glycemia-0', glycemia-60', glycemia-120' during oral glucose tolerance test, glycated-HbA1c, total-insulin, ultrasensitive CRP (C-reactive protein), liver steatosis by ultrasound. For the normal weight healthy control group, namely nwHD we considered a subselection of the above-mentioned clinical parameters, which are sex, age, BMI and BMI-SDS. The red and blu colors in the WGCNA heatmaps indicate respectively the identified positive or negative Pearson correlations: a positive correlation means that the abundance increase of that specific cluster(s) of taxa is associated with the increase of a specific clinical or metabolic parameter while a negative correlation is associated with a decrease of that specific clinical or metabolic parameter. Furthermore, for the species of OB-G and OBc-G samples groups we proceed with the analysis of the complete data set, but also with a manual selection of the species (indicated by the label “sel-specie”), namely a feature reduction, considering only those species that we found relevant in both supervised random forest and relative abundance analyses. The metagenome functional content was predicted using PICRUSt2^63^ from the biom file, to get the KEGG Orthology (KO) terms table and the inferred MetaCycpathways^18^. These data were analyzed with the Shotgun-data-profiling module of Microbiome Analyst to identify a list of the most significant pathways able to discriminate cases (OB-G or OBc-G) from controls (nwHD). The abundance of the pathways between the groups was also analyzed with the Wilcoxon test and the statistically significant pathways were clustered, considering the Pearson correlation measureand plotted using Morpheus tool (Morpheus, https://software.broadinstitute.org/morpheus).

### Informed consent

Informed consent was obtained from all individual participants or their families included in the study.

## Supplementary Information

Supplementary Information 1.

Supplementary Information 2.

## Data Availability

Raw 16S rRNA gene reads were deposited at the short read archive (SRA_BioProject ID PRJNA794317).
