# Supplementary material for: Gut-microbiota in children and adolescents with obesity: inferred functional analysis and machine-learning algorithms to classify microorganisms
Source: Sci Rep. 2023 Jul 12;13:11294. doi: 10.1038/s41598-023-36533-2 (PMC10338520; doi:10.1038/s41598-023-36533-2)
Supplement: Supplementary file 1 — Supplementary Information 1. [file 41598_2023_36533_MOESM1_ESM.pdf]

Supplementary Figure 1. **Alpha and beta diversity analyses.**

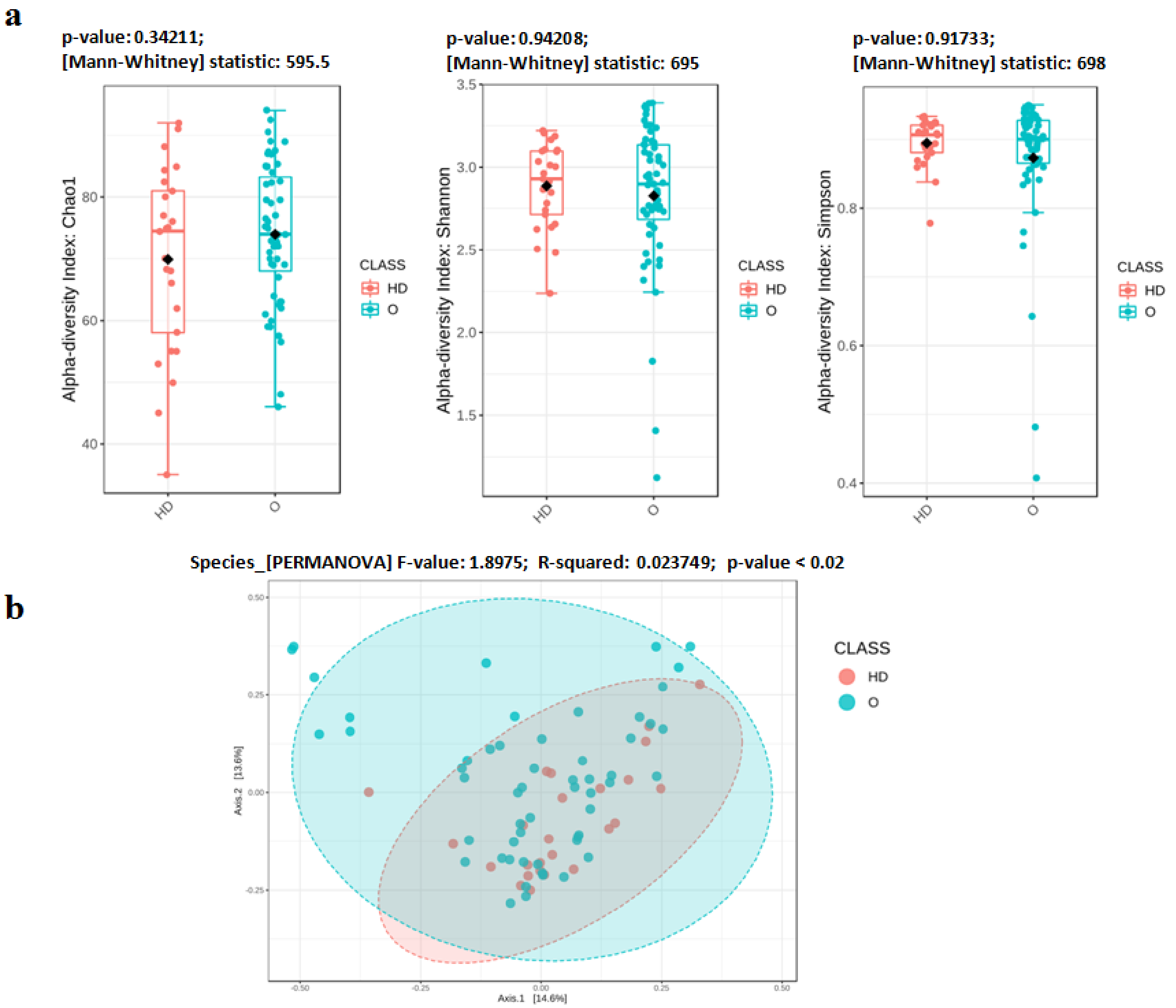

Supplementary Figure 1 (a) alpha diversity resultscomparing the complete cohort of Obese patients (fullOB), either without or with complications, with normal weight donors subjects (HD), 55 and 25 individuals, respectively. From left to right indexes estimate the community richness (Chao1-index) or community richness and evenness (Shannon or Simpson indexes). **(b)** The beta diversity result, on the same group comparison performed with the Principal Coordinates Analysis (PCoA) and based on Bray–Curtis distance matrix,showed that the microbial communities characterizing fullOB and HD donors are distinct. Permutational Multivariate Analysis of Variance (PERMANOVA) was used for statistical significance analysis.
