# Supplementary material for: Gut-microbiota in children and adolescents with obesity: inferred functional analysis and machine-learning algorithms to classify microorganisms
Source: Sci Rep. 2023 Jul 12;13:11294. doi: 10.1038/s41598-023-36533-2 (PMC10338520; doi:10.1038/s41598-023-36533-2)
Supplement: Supplementary file 2 — Supplementary Information 2. [file 41598_2023_36533_MOESM2_ESM.pdf]

Supplementary Table 1. Microbiome relative abundance analysis on the complete cohort of obese patients (fullOB) compared to normal weight Healthy Donors (nwHD) samples.

**fullOB [55] *vs*nwHD [25]**	**zero-inflated Gaussian fit**	**EdgeR**		**DESeq2**		**LDA**	
**Higher abundance in Obese patient samples**	FDR	log2FC	FDR	log2FC	FDR	LDA-SCORE	FDR
p_Bacteroidetes; c_Bacteroidia; o_Bacteroidales; f_Bacteroidaceae; ***g_Bacteroides; s_massiliensis***	0.0073						
p_Bacteroidetes; c_Bacteroidia; o_Bacteroidales; f_Bacteroidaceae; ***g_Bacteroides; s_faecis***	1.06E-4	2.8918	0.0018				
p_Bacteroidetes; c_Bacteroidia; o_Bacteroidales; f_Bacteroidaceae; ***g_Bacteroides; s_fragilis***		2.6643	0.0386				
p_Bacteroidetes; c_Bacteroidia; o_Bacteroidales; f_Prevotellaceae; ***g_Prevotella***		3.3504	0.0307				
p_Bacteroidetes; c_Bacteroidia; o_Bacteroidales; f_Prevotellaceae; ***g_Prevotella; s_copri***		3.3511	0.0387				
p_Firmicutes; c_Clostridia; o_ Clostridiales; f_ Lachnospiraceae; ***g_Coprococcus; s_catus***		1.5859	0.0230				
p_Firmicutes; ***c_Negativicutes***	0.0019	1.0488	0.0371	1.0867	0.0058	3.57	0.0015
p_Firmicutes; c_Negativicutes; ***o_Selenomonadales***	8.31E-4			1.0269	0.0212	3.57	0.0019
p_Firmicutes; c_Negativicutes; o_Selenomonadales; f_Acidaminococcaceae; ***g_Acidaminococcus***	7.08E-13	6.0228	8.34E-6	11.842	1.70E-9		
p_Firmicutes; ***c_Bacilli***				0.9733	0.0337	3.21	0.0405
p_Firmicutes; c_Bacilli; ***o_Lactobacillales***				1.0144	0.0493		
p_Firmicutes; c_Bacilli; o_Lactobacillales; ***f_Carnobacteriaceae***	0.0146						
*p_Firmicutes; c_Bacilli; o_Lactobacillales; f_Lactobacillaceae;****g_Lactobacillus***	0.0212						
p_Firmicutes; c_Bacilli; o_Lactobacillales; f_Streptococcaceae; ***g_Streptococcus***		2.0756	0.0080	2.0341	7.22E-4		
p_Firmicutes; c_Bacilli; o_Lactobacillales; f_Streptococcaceae; ***g_Streptococcus; s_thermophilus***		3.7707	6.0E-4	3.695	0.0478		
p_Proteobacteria; c_Betaproteobacteria; o_Burkholderiales; f_Sutterellaceae; ***g_Sutterella***	0.0027	3.7095	7.94E-4	3.156	0.01625	3.3	0.0412
p_Proteobacteria; c_Betaproteobacteria; o_Burkholderiales; f_Sutterellaceae; ***g_Sutterella; s_wadsworthensis***	0.0278	3.4795	0.0013	3.1663	0.0478		
p_Synergistetes; c_Synergistia; o_Synergistales; ***f_Synergistaceae***	0.0145						
**Higher abundance in normal weight Healthy Donor (nwHD) samples**							
p_Actinobacteria; c_Actinobacteria; o_Bifidobacteriales; f_Bifidobacteriaceae; ***g_Bifidobacterium; s_longum***	0.0051	-2.6775	6.71E-7	-1.636	0.0478		
p_Bacteroidetes; c_Bacteroidia; o_Bacteroidales; f_Bacteroidaceae; ***g_Bacteroides; s_eggerthii***		-2.2833	0.0387				
p_Bacteroidetes; c_Bacteroidia; o_Bacteroidales; ***f_Rikenellaceae***	0.0145						
*p_Bacteroidetes; c_Bacteroidia; o_Bacteroidales; f_Rikenellaceae;****g_Alistipes***	0.0027						
*p_Bacteroidetes; c_Bacteroidia; o_Bacteroidales; f_Rikenellaceae;****g_Alistipes; s_finegoldii***		-1.4927	0.0387				
*p_Bacteroidetes; c_Bacteroidia; o_Bacteroidales; f_Rikenellaceae;****g_Alistipes; s_senegalensis***	0.0376						
*p_Bacteroidetes; c_Bacteroidia; o_Bacteroidales; f_Rikenellaceae;****g_Alistipes; s_sp.***		-1.6049	0.0315				
p_Bacteroidetes; c_Bacteroidia; o_Bacteroidales; f_Porphyromonadaceae; ***g_Barnesiella***	0.0435						
p_Bacteroidetes; c_Bacteroidia; o_Bacteroidales; f_Porphyromonadaceae; ***g_Odoribacter***	0.0428						
p_Firmicutes; c_Clostridia; o_ Clostridiales; f_Eubacteriaceae; ***g_Eubacterium; s_hallii***	0.0278	-2.1665	4.29E-5				
*p_Firmicutes; c_Clostridia; o_ Clostridiales;****f_Gracilibacteraceae***	0.0025						
p_Firmicutes; c_Clostridia; o_ Clostridiales; ***f_Peptococcaceae***	0.0102						
p_Firmicutes; c_Clostridia; o_Clostridiales; f_Lachnospiraceae; ***g_Blautia; s_faecis***	0.0137						
p_Firmicutes; c_Clostridia; o_Clostridiales; f_unclassifiedClostridiales; ***g_Pseudoflavonifractor***	5.88E-6	-3.3663	4.39E-7				
p_Firmicutes; c_Clostridia; o_Clostridiales; f_Ruminococcaceae; ***g_Ruminiclostridium***	0.0016						
p_Firmicutes; c_Clostridia; o_Clostridiales; f_Ruminococcaceae; ***g_Ruminococcus; s_flavefaciens***	0.0298						
p_Firmicutes; ***c_Erysipelotrichia***	0.0248						
p_Firmicutes; c_Erysipelotrichia; o_Erysipelotrichales; ***f_Erysipelotrichaceae***	0.0145						
p_Firmicutes; c_Negativicutes; o_Selenomonadales; f_Veillonellaceae; ***g_Veillonella***	0.0219	-3.0716	0.0012				
p_Firmicutes; c_Negativicutes; o_Selenomonadales; f_Veillonellaceae; ***g_Veillonella; s_alcalescens***	0.0034	-4.5709	1.68E-7				
p_Firmicutes; c_Negativicutes; o_Selenomonadales; f_Veillonellaceae; ***g_Veillonella; s_rogosae***	0.0172	-1.974	0.0230				

p_Proteobacteria; c_Betaproteobacteria; o_Burkholderiales; f_Sutterellaceae; ***g_Parasutterella***	0.0025						
p_Verrucomicrobia; c_ Verrucomicrobiae; o_ Verrucomicrobiales; f_ Verrucomicrobiaceae; ***g_Akkermansia***	0.0025						
p_Verrucomicrobia; c_ Verrucomicrobiae; o_ Verrucomicrobiales; f_ Verrucomicrobiaceae; ***g_Akkermansia; s_muciniphila***	0.0376						

The number in square brackets indicates the number of patients belonging to the groups compared in the analysis. The differential abundance statistical analysis for microbial marker-gene used different “metagenomeSeq” and “RNASeq” methods. The “metagenomeSeq” use the zero-inflated Gaussian Fit algorithm. RNASeqis a differential abundance analysis method following EdgeR or DESeq2 algorithms. All statistical analysis adjust the data for imbalanced class distribution (under-sampling) and sparsity of a dataset. The taxa have been organized as *p_Phylum; c_Class; o_Order; f_Family; g_Genus; s_Specie*. FDR (False Discovery Rate) indicates the statistical significance p-value after adjustment for multiple comparisons. The base 2 logarithmic value of fold changes (log2FC) represents how much is an increase or decrease in abundance of a particular taxon in the comparisons between the indicated group of samples. A positive number indicates a higher abundance in the Obese group (OB-G) of samples, while negative values indicated the preferential abundance innwHD samples. FDR equal to or less than 0.05 was considered statistically significant.

Supplementary Table 2. Microbiome relative abundance analysis on simple Obese (OB-G) patients *vs*normal weight healthy donor (nwHD).

**Phylum**	**Simple Obese (OB-G) [34]<br>*vs*<br>normal weight healthy donor (nwHD) [25]**	**zero-inflated Gaussian fit**	**EdgeR**		**DESeq2**		**LDA**	
		FDR	log2FC	FDR	log2FC	FDR	LDA - SCORE	FDR
**Higher abundance in Obese (OB-G) patient samples**								
p_Actinobacteria; c_Actinobacteria; o_Coriobacteriales; f_Coriobacteriaceae; ***g_Collinsella; s_aerofaciens***			2.3571	0.0144				
p_Bacteroidetes; c_Bacteroidia; o_Bacteroidales; f_Bacteroidaceae; ***g_Bacteroides; s_faecis***		0.0017	3.0817	0.0041				
p_Bacteroidetes; c_Bacteroidia; o_Bacteroidales; f_Bacteroidaceae; ***g_Bacteroides; s_fragilis***			3.7523	0.0033				
p_Bacteroidetes; c_Bacteroidia; o_Bacteroidales; f_Bacteroidaceae; ***g_Bacteroides; s_plebeius***		0.0189						
p_Bacteroidetes; c_Bacteroidia; o_Bacteroidales; f_Bacteroidaceae; ***g_Bacteroides; s_thetaiotaomicron***			2.1903	0.0239				
p_Bacteroidetes; c_Bacteroidia; o_Bacteroidales; f_Porphyromonadaceae; ***g_Butyricimonas; s_virosa***		0.0363						
p_Bacteroidetes; c_Bacteroidia; o_Bacteroidales; f_Prevotellaceae; ***g_Prevotella***			3.3225	0.0311				
p_Bacteroidetes; c_Bacteroidia; o_Bacteroidales; f_Prevotellaceae; ***g_Prevotella; s_copri***		0.0194						
p_Firmicutes; ***c_Bacilli***					1.2083	0.0106	3.32	0.0114
p_Firmicutes; c_Bacilli; ***o_Lactobacillales***					1.3449	0.0108	3.32	0.0172
p_Firmicutes; c_Bacilli; o_Lactobacillales;f_Lactobacillaceae; ***g_Lactobacillus***		0.0195						
*p_Firmicutes; c_Bacilli; o_Lactobacillales;****f_Streptococcaceae***			2.1523	0.0184	1.9386	0.0156		
p_Firmicutes; c_Bacilli; o_Lactobacillales;f_Streptococcaceae; ***g_Streptococcus***			2.6028	0.0014	2.3288	0.0014		
p_Firmicutes; c_Bacilli; o_Lactobacillales;f_Streptococcaceae; ***g_Streptococcus; s_australis***		0.0047	2.4211	0.0137				
p_Firmicutes; c_Bacilli; o_Lactobacillales;f_Streptococcaceae; ***g_Streptococcus; s_salivarius***			2.1653	0.0137				
p_Firmicutes; c_Bacilli; o_Lactobacillales;f_Streptococcaceae; ***g_Streptococcus; s_thermophilus***		9.99E-4	5.0708	2.29E-4				
p_Firmicutes; c_Clostridia; o_Clostridiales; f_Lachnospiraceae; ***g_Coprococcus; s_catus***		0.0363	2.3273	0.0086				
p_Firmicutes; c_Clostridia; o_Clostridiales; f_Lachnospiraceae; ***g_Coprococcus; s_comes***			2.1999	0.0239				
*p_Firmicutes; c_Clostridia; o_Clostridiales; f_Ruminococcaceae;****g_Ruminococcus; s_flavefaciens***		0.0021	2.2387	0.0474				
p_Firmicutes; ***c_Negativicutes***							3.54	0.0143
*p_Firmicutes; c_Negativicutes;****o_Selenomonadales***							3.54	0.0179
p_Firmicutes; c_Negativicutes; o_Selenomonadales; f_Acidaminococcaceae; ***g_Acidaminococcus***		7.23E-9	4.6898	2.45E-4				
p_Proteobacteria; c_Betaproteobacteria; o_Burkholderiales; f_Sutterellaceae; ***g_Sutterella***		0.0116	3.7642	0.0011	3.4533	0.0403		
p_Proteobacteria; c_Betaproteobacteria; o_Burkholderiales; f_Sutterellaceae; ***g_Sutterella; s_stercoricanis***		1.07E-5	4.1523	0.0022				
p_Proteobacteria; c_Betaproteobacteria; o_Burkholderiales; f_Sutterellaceae; ***g_Sutterella; s_wadsworthensis***		9.99E-4	4.6938	2.93E-4				
p_Proteobacteria; c_Deltaproteobacteria; o_Desulfovibrionales; f_Desulfovibrionaceae; ***g_Desulfovibrio***		0.0096						
*p_Verrucomicrobia;c_Verrucomicrobiae;o_Verrucomicrobiales; f_Verrucomicrobiaceae;****g_Akkermansia;***		0.0064						
**Higher abundance in normal weight healthy donor (nwHD) patient samples**								
p_Actinobacteria; c_Actinobacteria; o_Actinobacteriales; f_Bifidobacteriaceae; ***g_Bifidobacterium; s_longum***		9.99E-4	-2.095	0.0133				
p_Bacteroidetes; c_Bacteroidia; o_Bacteroidales; f_Porphyromonadaceae; ***g_Barnesiella***		0.0096						
p_Bacteroidetes; c_Bacteroidia; o_Bacteroidales; f_Porphyromonadaceae; ***g_Odoribacter***		0.0345						
p_Bacteroidetes; c_Bacteroidia; o_Bacteroidales; ***f_Rikenellaceae***		0.0039						
*p_Bacteroidetes; c_Bacteroidia; o_Bacteroidales; f_Rikenellaceae;****g_Alistipes***		0.0015						
*p_Bacteroidetes; c_Bacteroidia; o_Bacteroidales; f_Rikenellaceae;****g_Alistipes; s_indistinctus***			-2.3560	0.0263				
*p_Bacteroidetes; c_Bacteroidia; o_Bacteroidales; f_Rikenellaceae;****g_Alistipes; s_senegalensis***		0.0025						
*p_Firmicutes; c_Clostridia; o_Clostridiales;****f_Christensenellaceae***		0.0490						
p_Firmicutes; c_Clostridia; o_Clostridiales; f_Eubacteriaceae; ***g_Eubacterium; s_siraeum***		9.99E-4						
p_Firmicutes; c_Clostridia; o_Clostridiales; f_Eubacteriaceae; ***g_Eubacterium; s_hallii***		0.0021	-1.8187	0.0263				
*p_Firmicutes; c_Clostridia; o_Clostridiales;****f_Gracilibacteraceae***		0.0039						

p_Firmicutes; c_Clostridia; o_Clostridiales; ***f_Peptococcaceae***	0.0016						
p_Firmicutes; c_Clostridia; o_Clostridiales; f_unclassifiedClostridiales; ***g_Pseudoflavonifractor***	4.80E-5	-3.5767	8.51E-5				
p_Firmicutes; c_Clostridia; o_Clostridiales; ***f_Ruminococcaceae***	0.0301						
p_Firmicutes; c_Clostridia; o_Clostridiales; f_Ruminococcaceae; ***g_Ruminiclostridium***	0.0064						
p_Firmicutes; c_Clostridia; o_Clostridiales; f_Ruminococcaceae; ***g_Ruminiclostridium; s_siraeum***	8.24E-4	-2.5699	0.0033				
p_Firmicutes; c_Clostridia; o_Clostridiales; f_Ruminococcaceae; ***g_Ruminococcus; s_lactaris***	0.0048	-2.8781	0.0029				
p_Firmicutes; c_ Erysipelotrichia; o_ Erysipelotrichales; ***f_Erysipelotrichaceae***	0.0201						
p_Firmicutes; c_ Erysipelotrichia; o_ Erysipelotrichales; f_Erysipelotrichaceae; ***g_[Eubacterium]***		-2.5252	0.0399				
p_Firmicutes; c_Negativicutes;o_Selenomonadales; f_Veillonellaceae; ***g_Veillonella***		-2.9261	0.0147				
p_Firmicutes; c_Negativicutes;o_Selenomonadales; f_Veillonellaceae; ***g_Veillonella; s_alcalescens***		-3.6547	0.0033				
p_Proteobacteria; c_Betaproteobacteria; o_Burkholderiales; ***f_Oxalobacteraceae***	0.0201						
p_Proteobacteria; c_Betaproteobacteria; o_Burkholderiales; f_Oxalobacteraceae; ***g_Herbaspirillum***	1.18E-5						
p_Proteobacteria; c_Betaproteobacteria; o_Burkholderiales; f_Sutterellaceae; ***g_Parasutterella***	0.0015						
p_Synergistetes; c_Synergistia; o_Synergistales; ***f_Synergistaceae***	0.0301						

The number in square brackets indicates the number of patients belonging to the groups compared in the analysis. The differential abundance statistical analysis for microbial marker-gene used different “metagenomeSeq” and “RNASeq” methods. The “metagenomeSeq” use the zero-inflated Gaussian Fit algorithm. RNASeqis a differential abundance analysis method following EdgeR or DESeq2 algorithms. All statistical analysis adjust the data for imbalanced class distribution (under-sampling) and sparsity of a dataset. The taxa have been organized as *p_Phylum; c_Class; o_Order; f_Family; g_Genus; s_Specie*. FDR (False Discovery Rate) indicates the statistical significance p-value after adjustment for multiple comparisons. The base 2 logarithmic value of fold changes (log2FC) represents how much is an increase or decrease in abundance of a particular taxon in the comparisons between the indicated group of samples. A positive number indicates a higher abundance in the Obese group (OB-G) of samples, while negative values indicated the preferential abundance innwHD samples. FDR equal to or less than 0.05 was considered statistically significant.

Supplementary Table 3. Microbiome relative abundance analysis on the group of patients Obese with complication (OBc-G) *vs* normal weight donor samples (nwHD).

**Obese with complication (OBc-G) [21]** ***vs*** **normal weight donor samples (nwHD) [25]**	**zero-inflated Gaussian fit**	**EdgeR**		**DESeq2**		**LDA**	
	FDR	log2FC	FDR	log2FC	FDR	LDA - SCORE	FDR
**Higher abundance in Obese with complication (OBc-G) patient samples**							
*p_Bacteroidetes; c_Bacteroidia; o_Bacteroidales; f_Bacteroidaceae; **g_Bacteroides; s_faecis***	0.0201	3.1409	0.0098				
*p_Bacteroidetes; c_Bacteroidia; o_Bacteroidales; f_Bacteroidaceae; **g_Bacteroides; s_massiliensis***	7.09E-4	2.9527	0.0433			3.13	0.0450
*p_Bacteroidetes; c_Bacteroidia; o_Bacteroidales; f_Prevotellaceae; **g_Prevotella***		3.7841	0.0211				
*p_Bacteroidetes; c_Bacteroidia; o_Bacteroidales; f_Prevotellaceae; **g_Prevotella; s_copri***		4.4218	0.0182				
*p_Firmicutes; c_Clostridia; o_Clostridiales; f_Lachnospiraceae; **g_Coprococcus; s_comes***						2.16	0.0450
*p_Firmicutes; c_Clostridia; o_Clostridiales; f_Lachnospiraceae;**g_Pseudobutyrivibrio; s_ruminis***		2.7618	0.0445				
*p_Firmicutes; **c_Negativicutes***	0.0066			1.2655	0.0069	3.63	0.0019
*p_Firmicutes; c_Negativicutes; **o_Selenomonadales***	0.0058			1.2416	0.0163	3.63	0.0023
*p_Firmicutes; c_Bacilli; o_Lactobacillales; f_Streptococcaceae; **g_Streptococcus; s_thermophilus***		2.4915	0.0433				
*p_Proteobacteria; c_Betaproteobacteria; o_Burkholderiales; f_Sutterellaceae; **g_Sutterella***		2.9930	0.0211				
*p_Proteobacteria; c_Betaproteobacteria; o_Burkholderiales; f_Sutterellaceae; **g_Sutterella; s_wadsworthensis***		4.1985	0.0046				
**Higher abundance in normal weight donor (nwHD) samples**							
*p_Bacteroidetes; c_Bacteroidia; o_Bacteroidales; f_Bacteroidaceae; **g_Bacteroides; s_ovatus***		-2.7690	0.0155				
*p_Firmicutes; c_Clostridia; o_ Clostridiales; f_Clostridiaceae; **g_Clostridium; s_lavalense***		-2.0408	0.0433				
*p_Firmicutes; c_Clostridia; o_ Clostridiales; f_ Lachnospiraceae; **g_Roseburia; s_faecis***		-1.8549	0.0445				
*p_Firmicutes; c_Clostridia; o_Clostridiales; f_Eubacteriaceae; **g_Eubacterium; s_siraeum***	0.0201						
*p_Firmicutes; c_Clostridia; o_Clostridiales; f_Eubacteriaceae; **g_Eubacterium; s_hallii***		-3.1492	0.0057				
*p_Firmicutes; c_Clostridia; o_Clostridiales; f_Lachnospiraceae; **g_Blautia; s_faecis***		-2.3831	0.0107				
*p_Firmicutes; c_Clostridia; o_Clostridiales; f_Lachnospiraceae; **g_Blautia; s_wexlerae***		-3.1199	0.0057				
*p_Firmicutes; c_Clostridia; o_Clostridiales; f_unclassifiedClostridiales; **g_Pseudoflavonifractor***	0.0032	-2.6848	0.0211				
*p_Firmicutes; c_Clostridia; o_Clostridiales; f_Ruminococcaceae; **g_Ruminiclostridium***	0.0331	-2.8668	0.0211				
*p_Firmicutes; c_Clostridia; o_Clostridiales; f_Ruminococcaceae; **g_Ruminococcus; s_sp.***		-2.8159	0.0090				
*p_Firmicutes; c_Negativicutes; o_Selenomonadales; f_Veillonellaceae; **g_Veillonella; s_alcalescens***		-3.7351	0.0433				
*p_Proteobacteria; c_Betaproteobacteria; o_Burkholderiales; f_Oxalobacteraceae; **g_Herbaspirillum***	0.0034						

The number in square brackets indicates the number of patients belonging to the groups compared in the analysis. The differential abundance statistical analysis for microbial marker-gene used different “metagenomeSeq” and “RNASeq” methods. The “metagenomeSeq” use the zero-inflated Gaussian Fit algorithm. RNASeqis a differential abundance analysis method following EdgeR or DESeq2 algorithms. All statistical analysis adjust the data for imbalanced class distribution (under-sampling) and sparsity of a dataset. The taxa have been organized as *p_Phylum; c_Class; o_Order; f_Family; g_Genus; s_Specie*. FDR (False Discovery Rate) indicates the statistical significance p-value after adjustment for multiple

comparisons. The base 2 logarithmic value of fold changes (log2FC) represents how much is an increase or decrease in abundance of a particular taxon in the comparisons between the indicated group of samples. A positive number indicates a higher abundance in the Obese with complication group (OBc-G) of samples, while negative values indicated the preferential abundance innwHD samples. FDR equal to or less than 0.05 was considered statistically significant.

Supplementary Table 4: Supervised analysis _ Random Forest.

Taxa	Group of correlation	OBB error	Sensitivity	Specificity	Meandecreaseaccuracy
	**fullOB [55] *vs* nwHD [25]**				
Streptococcus	fullOB	0.288	0.63	0.72	0.012
Lactobacillus	fullOB				0.008
Sutterella	fullOB				0.007
Doreaformicigenerans	fullOB	0.262	1.00	0.72	0.006
Alistipesonderdonkii	nwHD				0.006
Sutterellawadsworthensis	fullOB				0.005
Lactobacillusrogosae	fullOB				0.005
Coprococcuscomes	fullOB				0.004
	**Overall Severe Obese [32] *vs* nwHD [25]**				
Lactobacillus	Severe Obese	0.386	0.57	0.65	0.019
Sutterella	Severe Obese				0.013
Akkermansia	nwHD				0.007
Clostridium	Severe Obese				0.006
Streptococcus	Severe Obese				0.005
Acidaminococcus	Severe Obese				0.004
Bilophila	Severe Obese				0.003
Lactobacillusrogosae	Severe Obese	0.281	0.70	0.74	0.011
Sutterellawadsworthensis	Severe Obese				0.011
Blautiaproducta	Severe Obese				0.008
Bilophilawadsworthia	Severe Obese				0.006
Akkermansiamuciniphila	nwHD				0.006
Blautiawexlerae	nwHD				0.004
	**OB-G [34] *vs* nwHD [25]**				
Streptococcus	OB-G	0.322	0.64	0.70	0.018
Sutterella	OB-G				0.016
Clostridium	OB-G				0.013
Lactobacillus	OB-G				0.005
Alistipesonderdonkii	nwHD	0.356	0.61	0.66	0.009
Sutterellawadsworthensis	OB-G				0.005
Bifidobacteriumlongum	nwHD				0.005
Blautiaproducta	OB-G				0.005
	**OBc-G [21] *vs* nwHD [25]**				
Lactobacillus	OBc-G	0.413	0.61	0.56	0.012
Gemminger	OBc-G				0.009
Bacteroides	OBc-G				0.005
Oscillibacter	OBc-G				0.005
Sutterella	OBc-G				0.005
Coprococcuscomes	OBc-G	0.37	0.64	0.61	0.015
Bacteroidesmassiliensis	OBc-G				0.010
	**OB-SO-G [22] *vs* nwHD [25]**				
Lactobacillus	OB-SO-G	0.362	0.63	0.65	0.019
Sutterella	OB-SO-G				0.019
Bifidobacteriumlongum	nwHD	0.319	0.65	0.77	0.011
Sutterellawadsworthensis	OB-SO-G				0.009
Blautiaproducta	OB-SO-G				0.009
Lactobacillusrogosae	OB-SO-G				0.006
Alistipesonderdonkii	nwHD				0.005
	**OBc-SO-G[10] *vs* nwHD [25]**				
Lactobacillus	OBc-SO-G	0.229	0.77	0.75	0.016
Sutterella	OBc-SO-G				0.010
Parasutterella	nwHD				0.008
Haemophilus	nwHD				0.006
Oscillibacter	OBc-SO-G				0.006

Group of samples correlated for each taxa, out of bag (OBB) error, sensitivityand specificity of the test performed on different groups of samples are indicated on top of each comparison. The square bracketed number indicate the size of samples in each group used in the comparison. On the right the mean decrease accuracy values are indicated for each taxon, a higher value indicated a higher

correlation with the relative group of samples. "fullOB [55]": Complete casistic of obese patients; "Overall Severe Obese [32]": Complete casistics of Severely Obese patients; OB-G: group of simple obese [34] patients; OBc-G: group of obese with complication [21] patients; OB-SO-G [22]: A severely obese subgroup belonging to the Simple obese patients (OB-G); OBc-SO-G [10]: A severely obese subgroup part of the obese with complication patients group (OBc-G).

Supplementary Table 5. Unsupervised Analysis: WGCNA on Simple Obese (OB-G) [34], on Obese with complications (OBc-G) [21] and on normal weight Healthy Donors (nwHD) [25].

a) OBc-G (specie)

**Cluster 5: Cholesterol (positive, adj-p-value =0.04); LDL (positive, adj-p-value =0.01)**	
[1] "*Alistipesonderdonkii*"	*p_Bacteroidetes; c_Bacteroidia; o_Bacteroidales; f_Rikenellaceae; g_Alistipes; s_onderdonkii*
[2] "*Bacteroidesfaecichinchillae*"	*p_Bacteroidetes; c_Bacteroidia; o_Bacteroidales; f_ Bacteroidaceae; g_Bacteroides; s_faecichinchillae*
[3] "*Bacteroidesovatus"*	*p_Bacteroidetes; c_Bacteroidia; o_Bacteroidales; f_ Bacteroidaceae; g_Bacteroides; s_ovatus*
[4] "*Gordonibacterpamelaeae*"	*P_Actinobacteria; c_Actinobacteria; o_Coriobacteriales; f_ Coriobacteriaceae; g_Gordonibacter; s_ pamelaeae*
**Cluster 20: Glycem_0'(negative; adj-p-value =6.2E-04); Glycem_60' (negative; adj-p-value=0.03); Glycem_120' (negative; adj-p-value =0.01)**	
[1] "*Clostridiumcocleatum*"	*p_Firmicutes; c_Clostridia; o_Clostridiales; f_Clostridiaceae; g: Clostridium; s_cocleatum*
[2] "*Clostridiumramosum*"	*p_Firmicutes; c_Clostridia; o_Clostridiales; f_Clostridiaceae; g: Clostridium; s_ramosum*
**Cluster 21: Glycem_0'(negative; adj-p-value=9.0E-07); Glycem_60' (negative; adj-p-value=0.02); Glycem_120' (negative; adj-p-value= 1.3E-04)**	
[1] "*Acidaminococcusfermentans*"	*p_Firmicutes; c_Negativicutes; o_Selenomonadales; f_Acidaminococcaceae; g_Acidaminococcus; s_fermentans*
[2] "*Bifidobacteriumruminantium*"	*p_Actinobacteria*; c_*Actinobacteria; o_Bifidobacteriales; f_Bifidobacteriaceae; g_Bifidobacterium; s_ruminantium*
**Cluster 18: CRP (positive; adj-p-value =0.04)**	
[1] "*Alistipesindistinctus*"	*p_Bacteroidetes; c_Bacteroidia; o_Bacteroidales; f_Rikenellaceae; g_Alistipes; s_indistinctus*
[2] "*Bifidobacteriumpseudocatenulatum*"	p_*Actinobacteria*; c_*Actinobacteria; o_Bifidobacteriales; f_Bifidobacteriaceae; g_Bifidobacterium; s_pseudocatenulatum*
[3] "*Clostridiuminnocuum*"	*p_Firmicutes; c_Clostridia; o_Clostridiales; f_Clostridiaceae; g: Clostridium; s_innocuum*
[4] "*Desulfovibriopiger*"	*p_Proteobacteria; c_Deltaproteobacteria; o_Desulfovibrionales; f_Desulfovibrionaceae; g_Desulfovibrio; s_piger*
[5] "*Leuconostoccarnosum*"	*p_Firmicutes; c_Bacilli; o_Lactobacillales; f_Leuconostocaceae; g_Leuconostoc; s_carnosum*
[6] "*Mitsuokellajalaludinii*"	*p_Firmicutes; c_Negativicutes; o_Selenomonadales; f_Veillonellaceae; g_Mitsuokella; s_jalaludinii*
[7] "*Prevotellaruminicola"*	*p_Bacteroidetes; c_Bacteroidia; o_Bacteroidales; f_Prevotellaceae; g_Prevotella; s_ruminicola*
[8] "*Prevotella sp*."	*p_Bacteroidetes; c_Bacteroidia; o_Bacteroidales; f_Prevotellaceae; g_Prevotella; s_sp.*

b) OBc-G (sel-specie)

**Cluster 11: Glycem_0 (negative;adj-pvalue = 6.83E-06); Glycem_60 (negative;adj-pvalue 0.01);Glycem_120 (negative;adj-pvalue = 4.47E-05)**	
[1] "*Acidaminococcusfermentans*"	*p_Firmicutes; c_Negativicutes; o_Selenomonadales; f_Acidaminococcaceae; g_Acidaminococcus; s_fermentans*
[2] "*Clostridiumcocleatum*"	*p_Firmicutes; c_Clostridia; o_Clostridiales; f_Clostridiaceae; g: Clostridium; s_cocleatum*
[3] "*Clostridiumramosum*"	*p_Firmicutes; c_Clostridia; o_Clostridiales; f_Clostridiaceae; g: Clostridium; s_ramosum*
**Cluster 14: CRP (positive;adj-pvalue = 0.04)**	
[1] "*Alistipesindistinctus*"	*p_Bacteroidetes; c_Bacteroidia; o_Bacteroidales; f_Rikenellaceae; g_Alistipes; s_indistinctus*
[2] "*Bacteroidesxylanisolvens*"	*p_Bacteroidetes; c_Bacteroidia; o_Bacteroidales; f_ Bacteroidaceae; g_Bacteroides; s_xylanisolvens*
[3] "*Clostridiuminnocuum*"	*p_Firmicutes; c_Clostridia; o_Clostridiales; f_Clostridiaceae; g: Clostridium; s_innocuum*
[4] "*Clostridiumleptum*"	*p_Firmicutes; c_Clostridia; o_Clostridiales; f_Clostridiaceae; g: Clostridium; s_leptum*
[5] "*Desulfovibriopiger*"	*p_Proteobacteria; c_Deltaproteobacteria; o_Desulfovibrionales; f_Desulfovibrionaceae; g_Desulfovibrio; s_piger*
[6] "*Prevotellaruminicola*"	*p_Bacteroidetes; c_Bacteroidia; o_Bacteroidales; f_Prevotellaceae; g_Prevotella; s_ruminicola*
[7] "*Prevotellasp*"	*p_Bacteroidetes; c_Bacteroidia; o_Bacteroidales; f_Prevotellaceae; g_Prevotella; s_sp.*

c) OB-G (sel-specie)

**Cluster 3: Hb_gly (negative; adj-p-value 1.81E-5)**	
[6] "*Prevotellacopri*"	*p_Bacteroidetes; c_Bacteroidia; o_Bacteroidales; f_Prevotellaceae; g_Prevotella; s_copri*
[7] "*Prevotellasp*"	*p_Bacteroidetes; c_Bacteroidia; o_Bacteroidales; f_Prevotellaceae; g_Prevotella; s_sp.*
**Cluster 6: TRG (positive; adj-p-value = 0.04); Glycem_0' (negative; adj-p-value = 0.02)**	
[1] "*Clostridiumsaccharogumia*"	*p_Firmicutes; c_Clostridia; o_Clostridiales; f_Clostridiaceae; g: Clostridium; s_saccharogumia*
[2] "*Desulfovibriopiger* "	*p_Proteobacteria; c_Deltaproteobacteria; o_Desulfovibrionales; f_Desulfovibrionaceae; g_Desulfovibrio; s_piger*
**Cluster 19: LDL (negative;adj-p-value =0.02)**	
[1] "*Bacteroides clarus*"	*p_Bacteroidetes; c_Bacteroidia; o_Bacteroidales; f_ Bacteroidaceae; g_Bacteroides; s_clarus*
[2] "*Bacteroidesfaecichinchillae*"	*p_Bacteroidetes; c_Bacteroidia; o_Bacteroidales; f_ Bacteroidaceae; g_Bacteroides; s_faecichinchillae*
[3] "*Bacteroidesvulgatus*"	*p_Bacteroidetes; c_Bacteroidia; o_Bacteroidales; f_ Bacteroidaceae; g_Bacteroides; s_vulgatus*
[4] "*Bacteroidesxylanisolvens*"	*p_Bacteroidetes; c_Bacteroidia; o_Bacteroidales; f_ Bacteroidaceae; g_Bacteroides; s_xylanisolvens.*
[5] "*Collinsella intestinalis* "	*p_Actinobacteria;c_Actinobacteria; o_Coriobacteriales; f_Coriobacteriaceae; g_Collinsella; s_intestinalis*
[6] "*Sutterellaparvirubra*"	*p_Proteobacteria; c_ Betaproteobacteria; o_Burkholderiales; f_Sutterellaceae; g_Sutterella; s_ parvirubra*
[7] "*Sutterellawadsworthensis*"	*p_Proteobacteria; c_ Betaproteobacteria; o_Burkholderiales; f_Sutterellaceae; g_Sutterella; s_ wadsworthensis*

d) OB-G (family)

**Cluster 4: Glycem_0' (negative; adj-p-value =2.48E-09)**	
[1] "*Alteromonadaceae*"	*p_Proteobacteria; c_Gammaproteobacteria; o_Alteromonadales; f_Alteromonadaceae*
[2] "*Brucellaceae*"	*p_Proteobacteria; c_Alphaproteobacteria; o_Rhizobiales; f_Brucellaceae*
[3] "*Cohaesibacteraceae*"	*p_Proteobacteria; c_Alphaproteobacteria; o_Rhizobiales; f_Cohaesibacteraceae*
[4] "*Cyclobacteriaceae*"	*p_Bacteroidetes; c_Cytophagia; o_Cytophagales; f_Cyclobacteriaceae*
[5] "*Cytophagaceae*"	*p_Bacteroidetes; c_Cytophagia; o_Cytophagales; f_Cytophagaceae*
[6] "Gracilibacteraceae"	*p_Firmicutes; c_Clostridia; o_Clostridiales; f_Gracilibacteraceae*
[7] "*Haloplasmataceae*"	*p_unclassified bacteria; c_unclassified bacteria; o_Haloplasmatales; f_Haloplasmataceae*
[8] "*Kineosporiaceae*"	p_*Actinobacteria*; c_*Actinobacteria; o_Actinomycetales; f_Kineosporiaceae*
[9] "*Marinilabiliaceae*"	*p_Bacteroidetes; c_Bacteroidia; o_Bacteroidales; f_Marinilabiliaceae*
[10] "*Oxalobacteraceae*"	*p_Proteobacteria; c_ Betaproteobacteria; o_Burkholderiales; f_Oxalobacteraceae*
[11] "*Paenibacillaceae*"	*p_Firmicutes; c_Bacilli; o_Bacillales; f_Paenibacillaceae*
[12] "*Peptococcaceae*"	*p_Firmicutes; c_Clostridia; o_Clostridiales; f_Peptococcaceae*
[13] "*Pseudomonadaceae*"	*p_Proteobacteria; c_Gammaproteobacteria; o_Pseudomonadales; f_Pseudomonadaceae*
[14] "*Spiroplasmataceae*"	*p_Tenericutes; c_Mollicutes; o_Entomoplasmatales; f_Spiroplasmataceae*

e) OB-G (genus)

**Cluster 7: Glycem_0' (negative; adj-p-value =5.11E-05)**	
[1] "*Anaerostipes*"	*p_Firmicutes; c_Clostridia; o_Clostridiales; f_Lachnospiraceae; g_Anaerostipes*
[2] "*Butyricimonas*"	*p_Bacteroidetes; c_Bacteroidia; o_Bacteroidales; f_Porphyromonadaceae; g_Butyricimonas*
[3] "Coprobacillus"	*p_Firmicutes; c_Erysipelotrichia; o_Erysipelotrichales; f_Erysipelotrichaceae; g_Coprobacillus*
[4] "*Desulfovibrio*"	*p_Proteobacteria; c_Deltaproteobacteria; o_Desulfovibrionales; f_Desulfovibrionaceae; g_Desulfovibrio*
[5] "*Escherichia*"	*p_Proteobacteria; c_Gammaproteobacteria; o_Enterobacteriales; f_Enterobacteriaceae; g_Escherichia*
[6] "*Gardnerella*"	p_*Actinobacteria*; c_*Actinobacteria; o_Bifidobacteriales; f_Bifidobacteriaceae; g_Gardnerella*
[7] "*Herbaspirillum*"	*p_Proteobacteria; c_ Betaproteobacteria; o_Burkholderiales; f_Oxalobacteraceae; g_Herbaspirillum*
[8] "*Oxalobacter*"	*p_Proteobacteria; c_ Betaproteobacteria; o_Burkholderiales; f_Oxalobacteraceae; g_Oxalobacter*
[9] "*Pseudobutyrivibrio*"	*p_Firmicutes; c_Clostridia; o_Clostridiales; f_Lachnospiraceae; g_Pseudobutyrivibrio*
[10] "*Tannerella*"	*p_Bacteroidetes; c_Bacteroidia; o_Bacteroidales; f_Porphyromonadaceae; g_Tannerella*

f) nwHD (specie)

**Cluster 19: BMI-SDS (negative; adj-p-value =0.05)**	
[1] "*Bacteroides massiliensis*"	*p_Bacteroidetes; c_Bacteroidia; o_Bacteroidales; f_ Bacteroidaceae; g_Bacteroides; s_massiliensis*
[2] "*Collinsellaintestinalis*"	*p_Actinobacteria;c_Actinobacteria; o_Coriobacteriales; f_Coriobacteriaceae; g_Collinsella; s_intestinalis*
[3] "*Comamonaskerstersii*"	*p_Proteobacteria; c_Betaproteobacteria; o_Burkholderiales; f_Comamonadaceae; g_Comamonas; s_kerstersii*
[4] "*Lachnoclostridiumhathewayi*"	*p_Firmicutes; c_Clostridia; o_Clostridiales; f_Lachnospiraceae; g_Lachnoclostridium; s_hathewayi*
[5] "*Sutterellastercoricanis*"	*p_Proteobacteria;c_Betaproteobacteria; o_Burkholderiales; f_Sutterellaceae; g_Sutterella; s_stercoricanis*

Unsupervised Analysis: WGCNA. On the right column for each taxon is indicated the taxonomy: *p_Phylum; c_Class; o_Order; f_Family; g_Genus; s_Species*. Among round parenthesis is indicated the Pearson correlation (shown as negative/positive correlation), and on the right is the Benjamini Hochberg adjusted p-value of the correlation between the cluster of the taxa and the clinical variable. In the title among squared parenthesis is indicated the number of samples for each group. **(a)** Species (OBc-G specie) (16) from obese with complications (OBc-G) and the correlation with clinical variables. **(b-c)** Selected species (OBc-G sel-specie) (10) from obese with complications. The "sel-specie" indicates that before the WGCNA analysis we performed a feature reduction on the species considering those taxa that were found relevant within our previous analysis (see Methods section). **(d-e)** Selected families (14) and genera (10) from simple obese (OB-G) patient samples and the correlation with clinical variables. **(f)** Species (5) of normal weight HD donors and the correlation with BMI_SDS.
